# Balancing equity and policyholder protection: Assessing insurer’s interests in green lending under cap-and-trade regulations

**DOI:** 10.1371/journal.pone.0293975

**Published:** 2023-11-06

**Authors:** Shi Chen, Yonghong Zhao, Shiu-Chieh Chiu, Jingfei Wu, Jyh-Horng Lin

**Affiliations:** 1 Schools of Economics, Southwestern University of Finance and Economics, Chengdu, China; 2 Department of Banking and Finance, CTBC Business School, Tainan, Taiwan; 3 School of Economics, Shanghai University, Shanghai, China; 4 Department of International Business, Tamkang University, New Taipei City, Taiwan; Ovidius University of Constanta: Universitatea Ovidius din Constanta, ROMANIA

## Abstract

This paper presents a contingent claim model designed to assess an insurer’s equity within the framework of carbon trading regulations imposed on borrowing firms while also considering the integration of green lending. The development of this model is particularly relevant for regions with established carbon trading markets, with a specific focus on the post-period following the 2015 Paris Agreement concerning climate change. We focus on shareholders and policyholders to optimize equity and ensure maximum protection. Strict caps in cap-and-trade harm interest margins, reducing guaranteed rates for equity maximization and compromising policyholder protection. Government intervention through sustainable production carbon trading hinders win-win outcomes. Green subsidies can improve insurer margins, but achieving win-win solutions remains challenging. A collective approach is needed to share sustainable production and finance benefits among diverse economic sectors.

## 1. Introduction

Sustainability plays a crucial role in the insurance industry, with the Principles for Sustainable Insurance (PSI) established by the UNEP FI (United Nations Environment Programme Financial Initiative) as a prominent framework for achieving sustainability. These principles effectively address environmental, social, and governance (ESG) risks and opportunities, underscoring the industry’s dedication to sustainable practices, See UNEP Finance Initiative [1]. However, there is limited academic research on the relationship between ESG practices and stability in the insurance sector [2]. Moreover, existing literature primarily focuses on the impact of green finance on shareholder return maximization in borrowing firms’ sustainable production, overlooking the significant ESG ethos that prioritizes the needs of stakeholders [3, 4]. This paper aims to address the gaps in existing research by examining the impact of green finance on insurer performance in cap-and-trade transactions involving borrowing firms. By examining the perspective of borrowing firms engaged in carbon trading, we aim to shed light on how green finance influences insurer performance and contributes to sustainability from a broader stakeholder standpoint.

Based on a survey conducted by BearingPoint in collaboration with YouGov Germany, Hutschenreiter highlights that more than 70% of respondents expect insurance companies to promote sustainability through their products actively [5]. In life insurance, profit-sharing life insurance policies assume a significant role, where policyholder protection and participation levels are paramount. Furthermore, green finance serves as a crucial avenue for advancing sustainable development. Integrating firms’ green trades into the regulatory cap-and-trade mechanism can contribute to enhancing sustainability efficiency [4, 6]. Pursuing multiple objectives that align with environmental enhancement is imperative to achieve sustainable insurance operations.

Moreover, the Paris Agreement, which was adopted in December 2015, is a landmark international accord that addresses climate change. Fundamentally, the Paris Agreement’s focus on mitigating climate change, enhancing transparency, promoting adaptation, mobilizing climate finance, and fostering public-private collaboration provides a clear mandate for stakeholder engagement in the sustainable insurance sector. Insurers and other relevant stakeholders should align their strategies and operations with the goals of the agreement to effectively manage climate-related risks, support climate resilience, and contribute to a more sustainable and climate-friendly future. Hence, this paper underscores the pivotal role of stakeholder engagement in advancing diverse social initiatives, especially in the aftermath of the 2015 Paris Agreement, aimed at furthering sustainable development in alignment with the principles delineated in the PSI.

To contribute to the existing body of literature, we propose developing a contingent claim model for assessing an insurer’s equity and liabilities. Recognizing that numerous insurers operate within large-scale and imperfectly competitive loan and life insurance markets [7], our model emphasizes two key stakeholders within the insurance framework: shareholders and policyholders. Our primary objectives revolve around determining the optimal guaranteed rate to maximize equity and deciding the optimal loan rate to enhance policyholder protection. By addressing these crucial elements, we strive to foster sustainable production through carbon trading within the insurance sector.

The first aspect focuses on the insurer’s decision-making regarding the guaranteed rate, considering fixed loan rates, intending to maximize equity. As a financial institution, the insurer strives to optimize its equity to fulfill the fundamental functions of insurance, mainly when operating in an imperfectly competitive life insurance market. Equity maximization primarily benefits shareholders, who are among the key stakeholders of the insurer. The second aspect pertains to the insurer’s decision-making regarding loan rates while holding a constant guaranteed rate in asset-liability matching management. This decision-making approach aims to maximize policyholder protection. Higher returns on investment lead to increased protection for policyholders. Protection maximization primarily benefits policyholders, who are significant stakeholders of the insurer. Accordingly, our study will explore three “green” effects from the stakeholders’ perspective, including shareholders and policyholders. These three green effects encompass the regulatory cap of the cap-and-trade mechanism, green loan subsidies, and green loans. Through this investigation, we aim to contribute to the existing literature on green finance.

To fulfill our objective, we propose utilizing a contingent claim model that evaluates the equity value of an insurer, taking into account the influence of green subsidies and the involvement of borrowing firms partaking in cap-and-trade transactions within regulatory frameworks. Additionally, we incorporate the insurer’s liabilities as a metric for assessing policyholder protection. The insurer’s primary aim is to determine the optimal guaranteed rate, which maximizes the capped call option function denoted in terms of profits, thereby optimizing equity. Simultaneously, the insurer establishes the loan rate to ensure the highest level of policyholder protection. A comparative analysis can be conducted by integrating the optimization of equity holders with the maximization of policyholder protection.

One main finding reveals that stricter regulatory caps under the cap-and-trade mechanism lead to a lower guarantee rate (increased interest margin) for equity maximization and a lower loan rate (decreased margin) for policyholder protection. This regulatory cap affects the availability of life insurance policies for policyholders and loans for borrowing firms. Consequently, there is no win-win outcome among stakeholders regarding margins when stringent caps are imposed. Implementing ESG strategies for stakeholder benefit results in higher margins for the insurer. Second, government green subsidies impact guaranteed and loan rates, affecting margins. Win-win outcomes may be compromised. Green loan subsidies contribute to insurer margins in stakeholder-driven ESG management. Third, increasing green loans affects guaranteed and loan rates, impacting margins. This green policy challenges win-win outcomes in ESG management. Governments should participate in green trading, cap-and-trade regulations, and green technology subsidies to improve sustainability in the circular economy. Fourth, the total effects of regulatory caps and government green loan subsidies on sustainable insurance depend on stakeholder benefits. However, the results differ when it comes to green lending. These findings contribute to stakeholder engagement literature, addressing the need for clarity in business and society research [8].

ESG, as defined by the UNEP FI PSI, plays a vital role in stakeholder management. All stakeholders prioritize their interests, and insurers aim to achieve win-win outcomes by developing strategies that benefit everyone involved. However, it is essential to note that win-win outcomes are not always possible. In such cases, governments have a crucial role in supporting insurers and borrowers to promote sustainability in the circular economy by implementing green policies.

We organize the remaining part of the paper as follows. Section 2 introduces the recent literature review as the research ground background for the paper. Section 3 models the sustainable insurance theory. In Section 4, we solve the optimal guaranteed and loan rate for the maximum benefits for stakeholders and present comparative statics. Section 5 shows the comparative static results by using numerical analysis. The last section summarizes the main conclusions and puts forward implications.

## 2. Literature review and theoretical background

In order to establish the foundation for our research and elucidate the contribution of our paper, our study on sustainable insurance is aligned with three key strands of the literature.

### 2.1 Finance distribution and sustainability

First, the literature on finance distribution and sustainability in insurance addresses various aspects. For instance, Godínez-Olivares et al. propose a sustainability balancing mechanism for the pension system’s sustainability [9]. Schokkaert et al. discuss intragenerational equity through point distribution [10]. Romp and Beetsma examine intergenerational benefits in pension finance [11]. Fratoni et al. highlight the impact of COVID-19 on public pension schemes [12]. Huang and Lin find the cap-and-trade mechanism affects firms’ production and operation decisions and carbon emissions, making them move towards environmental sustainability [13].

The literature referenced previously underscores the vital importance of research at the intersection of finance distribution and sustainability. These studies collectively emphasize that sustainability in insurance goes beyond mere financial considerations. It encompasses social, economic, and environmental dimensions. In the face of evolving societal needs, global challenges like the COVID-19 pandemic, and the imperative to address climate change, research in this domain not only fills gaps in existing literature but also provides valuable insights for policymakers, industry professionals, and academics. It enables the development of insurance practices that are equitable, resilient, and capable of supporting both individuals and society as a whole in an ever-changing world.

While the existing body of research predominantly concentrates on matters of financial sustainability, our study offers a distinctive and invaluable perspective by actively bridging the existing gap in the literature. We delve into the unexplored realm of environmental sustainability within the context of green finance, with a particular focus on sustainable insurance. This emphasis is imperative in light of the escalating significance of environmental concerns within the financial sector, underscored by a pressing demand for comprehensive research in this pivotal area.

### 2.2 Green insurer finance

Second, the literature on green insurer finance examines various aspects. For instance, using a game theory model, Wang et al. study the impact of insurance subsidies on green insurance for sustainable development [14]. Chen et al. analyze the effects of green transactions by borrowing firms on insurer green finance through a contingent claims model, focusing on green technology options [3]. Li et al. investigate the relationship between borrowing-firm environmental quality and insurer green finance, considering green loan subsidies, cap-and-trade regulations, and green technology choices [4]. Zhou et al. find a positive link between green finance and environmental quality [15]. Bao and He find the role of green finance in adjusting the misallocation of financial resources and leading the green sustainable development of the real economy is increasingly important [16].

The literature mentioned earlier highlights the critical significance of research on green insurer finance. Collectively, this body of literature demonstrates that green insurer finance is not just a niche or isolated field; it is a critical driver of sustainable development. The findings have relevance for insurers, borrowers, policymakers, and investors. By emphasizing the role of finance in promoting environmental sustainability, these studies contribute to the broader understanding of how financial mechanisms can support or hinder sustainable practices, making a compelling case for the integration of green finance principles in the insurance industry and beyond.

While these studies primarily emphasize environmental sustainability within an equity-maximizing framework for green insurance, our analysis centers on environmental enhancements catalyzed by insurer green finance, with a strong focus on maximizing stakeholder benefits in alignment with the UNEP FI PSI. Our unique contribution lies in bridging the gap between environmental sustainability and stakeholder value, offering a perspective that combines green finance and sustainable insurance practices for the more significant benefit of all involved parties.

### 2.3 Stakeholder engagement

The stakeholder engagement literature constitutes the third strand of research. O’Riordan and Fairbrass emphasize stakeholder engagement as facilitating dialogue between organizations and stakeholders [17]. Various studies examine topics such as implicit and explicit corporate social responsibility [18], environmental and sustainability issues [19], and financial and operational performance [20]. Baah et al. find that regulatory stakeholder pressures positively influence adoption of green production practices, firm reputation, and financial and environmental performance [21]. Green production practices are also shown to have a significant positive impact on firm reputation and environmental performance. Ang et al. observe that corporate social responsibility (CSR) positively affects the financial performance of heavily polluting Chinese listed companies [22]. However, ownership concentration diminishes this positive impact, while ownership balance enhances the positive effect of CSR on corporate financial performance. Kujala et al. comprehensively review contemporary literature on stakeholder engagement [8].

The literature on stakeholder engagement is of paramount importance due to its multifaceted implications for organizations, society, and the environment. Overall, the stakeholder engagement literature above is a rich source of insights for organizations striving to align their operations with societal and environmental needs. It highlights the significance of inclusivity, transparency, and responsibility in business conduct. It demonstrates that stakeholder engagement is not just a moral or ethical imperative but a strategic one with the potential to drive financial success and sustainability.

The literature underlines the critical role of stakeholder engagement in shaping corporate decision-making, covering areas such as social responsibility, sustainability, and financial performance. It accentuates the favorable outcomes linked to green initiatives, bolstering the firm reputation and improving both financial and environmental performance. Our research makes a distinct contribution by extending this understanding to the context of insurer green finance and environmental sustainability, where we explore the active involvement of key stakeholders. Through our work, we aim to illuminate the synergistic potential of stakeholder engagement in achieving environmentally and financially sustainable outcomes for insurers and their stakeholders.

## 3. Sustainable insurance model

### 3.1 Conceptual framework

Our study examines a scenario involving a life insurer and a borrowing firm. As a large-scale entity, the insurer is a rate-setter for investment loans and a rate-setter for guaranteed rates in life insurance policies within the insurance business model. This structure implies that borrowing firms, which may consist of small and medium enterprises, act as rate-takers. The insurer adheres to the requirements of the PSI by incorporating ESG principles into its financial management system.

Considering ESG, the insurer formulates a green lending policy to promote environmentally-friendly practices. Under this policy, the borrowing firm must participate in the cap-and-trade mechanism. If the borrowing firm acts as a carbon allowance seller, it benefits from a lower loan rate than a carbon allowance buyer. This difference can be viewed as a government subsidy aimed at encouraging sustainability. The degree of sustainability, denoted as “greenness,” reflects the environmental and governance aspects of the insurer’s ESG framework.

Furthermore, at the core of the insurance model lies the evaluation, pricing, and transfer of risks on behalf of life insurance policyholders, along with the implementation a liability-driven strategy. The alternative objective of the insurance model may involve setting loans and guaranteed rates to maximize shareholder equity value or policyholder protection. These alternative objective settings contribute to the social aspect of the insurer’s ESG performance. Therefore, while the insurance model aligns with the principles outlined in the PSI, stakeholder engagement becomes crucial due to alternative objectives.

### 3.2 Theoretical model

We consider a one-period (*t*∈[0,1]) insurance model. At the beginning of the period, the three balance sheets demonstrate the framework as follows:

The insurer (i.e., the fund provider):

$$L_{G}+L_{C}+B=P+E=\beta (L_{G}+L_{C}+B)+E$$ (1)

The green borrowing firm (i.e., the carbon allowance seller):

$$A_{G}=L_{G}+E_{G}$$ (2)

The carbon-intensive borrowing firm (i.e., the carbon allowance buyer):

$$A_{C}=L_{C}+E_{C}$$ (3)

Eq (1) describes that the liabilities of life insurance policies (*P* = *β*(*L*_*G*_+*L*_*C*_+*B*) where *β* = the leverage) and the equity capital (*E* = (1−*β*)(*L*_*G*_+*L*_*C*_+*B*)) finance the investment portfolio, including the green loans *L*_*G*_ = *αL*, the carbon-intensive loans *L*_*C*_ = (1−*α*)*L*, and the liquid assets (*B*), where 0<*α*<1 presents the strategic loan distribution ratio and *L* is defined as the total loans issued by the insurer. The term *A*_*G*_ is the green borrowing-firm investment funded by the insurer’s green loans (*L*_*G*_) and equity capital (*E*_*G*_) in Eq (2). The balance sheet of Eq (3) represents the investment (*A*_*C*_) financed by the loans (*L*_*C*_) and equity capital (*E*_*C*_).

On the asset side of the balance sheet, the insurer faces two downward-sloping curves of green and carbon-intensive loans since we assume that the borrowing firms are small and medium enterprises. Thus, we have the features: the demand function for green loans is the expression *L*_*G*_ = *L*_*G*_(*R*_*G*_) with the condition *∂L*_*G*_(*R*_*G*_)/∂*R*_*G*_ = ∂*L*_*G*_(*R*_*G*_)/∂*R*_*C*_<0, where *R*_*G*_ = *R*_*C*_−*R*_*S*_, *R*_*C*_ = the carbon-intensive loan rate, and RS *R*_*S*_ = the government’s subsidy rate for the green loan. The government encourages financial institutions to provide green loans with subsidies such that the green loan rate is less than the carbon-intensive loan rate [23]. We demonstrate the demand function for the carbon-intensive loan as the form *L*_*C*_(*R*_*C*_) with the downward-sloping condition ∂*L*_*C*_/∂*R*_*C*_<0. The advantage of the loan function expression explains the green loan rate is lower than the carbon-intensive loan due to the government subsidy. The insurer can determine the optimal carbon-intensive loan rate for alternative optimizations, simplifying the model computation. On the liability side, the insurer issues a profit-sharing life insurance policy expressed by the form *β*(*L*_*G*_+*L*_*C*_(*R*_*C*_))*e*^*R*^ [24]. We also assume that the insurer faces an upward-sloping supply function of the life insurance policy where the insurer is a guaranteed rate-setter (*R*). Thus, the life insurance market is imperfectly competitive.

The insurance model captures the PSI by explicitly capping the credit risks from the borrowing firms. The credit risk treatments make the insurer model the insurer’s equity as a capped call option [25]. Toward that end, we model the two borrowing-firm assets varying continuously during the period based on a Brownian motion (GBM):

$$dV_{G}/V_{G}=\mu_{G}dt+\sigma_{G}dW_{G}$$ (4)

$$dV_{C}/V_{C}=\mu_{C}dt+(\sigma_{C}+\sigma_{C}^{2}/2)dW_{C}$$ (5)

where

$$V_{C}=(1+R_{C})A_{C}+(c_{C}-cap)A_{C}=[(1+R_{C})+(c_{C}-cap)](L_{C}(R_{C})+E_{C})$$

$$c_{G}<cap<c_{C}$$

Eq (4) demonstrates that the asset return (*V*_*G*_) is with the instantaneous drift (*μ*_*G*_), the instantaneous volatility, and a Wiener process (*W*_*G*_). In the cap-and-trade mechanism, the green borrowing firm benefits from selling excess carbon allowances where *cap* = the regulatory cap rate, *c*_*G*_ = the marginal cost of carbon emission (i.e., air pollution), and *c*_*G*_<*cap*. Thus, the green borrowing-firm total investment returns include the investment returns and the sales of extra carbon allowances. Eq (5) presents that the asset return (*V*_*C*_) is with the instantaneous drift (*μ*_*C*_), the instantaneous volatility ($\sigma_{C}+\sigma_{C}^{2}/2$), and a Wiener process (*W*_*C*_). The asset return consists of the investment revenues and the cost of carbon allowance purchases for production permission, where *c*_*C*_ = the marginal cost of carbon emission and *cap*<*c*_*C*_. We assume that the carbon-intensive borrowing firm suffers from the climate change risk captured by the quadratic form in our model. According to the PSI, the insurer must assess the physical risk of the sustainable insurance policy. Our model explicitly considers borrowing-firm credit risks to evaluate policyholder protection. Relative speaking, we only model the physical risk from the carbon-intensive borrowing firm. The modeling reflects the features of the ESG for policyholder protection.

We need to define the insurer’s equity capping information about Eqs (4) and (5) before proceeding with the analysis of the policyholder protection. We apply Dermine and Lajeri and specify the insurer’s equity as follows [25]:

$$S(V,P_{I})=CS(V,P_{I})-\delta CS(\beta V,P_{I})$$ (6)

where

$$P_{I}=\beta (L_{G}+L_{C}+B)e^{R}-(1+R_{B})B$$

$$A=(1+R_{G})A_{G}+(1+R_{C})A_{C}$$

$$CS(V,P_{I})=[VN(b_{1})-P_{I}e^{-R_{B}}N(b_{2})]-[VN(b_{3})-Ae^{-R_{B}}N(b_{4})]$$

$$CS(\beta V,P_{I})=[\beta VN(d_{1})-P_{I}e^{-R_{B}}N(d_{2})]-[\beta VN(d_{3})-Ae^{-R_{B}}N(d_{4})]$$

$$V=V_{G}+V_{C},\;dV/V=\mu dt+\sigma dW,\;\sigma =[\sigma_{G}+(\sigma_{C}+\sigma_{C}^{2}/2)]/2$$

$$b_{1}=\frac{\ln(V/P_{I})+R_{B}+\sigma^{2}/2}{\sigma },\;b_{2}=b_{1}-\sigma$$

$$b_{3}=\frac{\ln(V/A)+R_{B}+\sigma^{2}/2}{\sigma },\;b_{4}=b_{3}-\sigma$$

$$d_{1}=\frac{\ln(\beta V/P_{I})+R_{B}+\sigma^{2}/2}{\sigma },\;d_{2}=d_{1}-\sigma$$

$$d_{3}=\frac{\ln(\beta V/A)+R_{B}+\sigma^{2}/2}{\sigma },\;d_{4}=d_{3}-\sigma$$

In Eq (6), the term *V* is the total investment returns of the two borrowing firms (i.e., *V*_*G*_+*V*_*C*_). The investment returns are with the instantaneous drift *μ*. The corresponding instantaneous volatility (*σ*) consists of the additive volatilities of the green borrowing firm and the carbon-intensive borrowing firm. The term *P*_*I*_ is the insurer’s net obligations, the total payments to the policyholders net of the liquid-asset repayments. We define the rate *R*_*B*_ as the liquid-asset market rate and specify the term *A* as the loan repayments from the green and carbon-intensive borrowing firms. Eq (6) expresses the insurer’s equity as a capped call option in a hybrid position. The first term on the right-hand side is the insurer’s total equity value, explicitly considering the capped credit risks from the borrowing firms. The second term is the participation level of the profit-sharing life insurance policy where *δ* = the participation rate.

Given information about Eq (6), we can value the insurer’s liabilities (i.e., equivalently policyholder protection), We apply Dermine and Lajeri to formulate Eq (7) [25].:

$$PP(V,P_{I})=P_{I}e^{-R_{B}}-[P_{I}e^{-R_{B}}N(-b_{2})-VN(-b_{1})]+\delta [\beta VN(d_{1})-P_{I}e^{-R_{B}}N(d_{2})]$$ (7)

Based on Eqs (6) and (7), we present two different scenarios from a stakeholder perspective. First, the insurer determines the optimal guaranteed rate to maximize the equity for shareholders, holding the loan rate constant due to the dichotomous asset-liability management, according to Sealey [26]. Life insurance policies are an essential core business. Determining the guaranteed rate in an imperfectly competitive insurance market is in the business of protecting from risks for equity maximization. Second, insurers must invest the collected funds from policyholders to pay claims and benefits. The insurer is to set the optimal loan rate to maximize the protection for policyholders, holding the guaranteed rate constant. Investment is at the heart of providing life insurance policies to protect policyholders. Insurance Europe and Oliver Wyman demonstrate that profit-sharing life insurance policies have the following features [27]. Available investments affect the guarantee level for new policies, while profit sharing for existing policies depends on investment performance. The former is an equity maximization issue, and the latter is a policyholder protection problem. Accordingly, the insurer adopts two guaranteed and loan rate-setting strategies to achieve maximum benefits for stakeholders (i.e., shareholders and policyholders). The optimization considers stakeholders, meeting ESG.

### 3.3 Solutions and comparative statics

We can partially differentiate Eq (6) with the guaranteed rate for equity maximization. The first-order condition is ∂*S*(*V*,*P*_*I*_)∂/*R* = 0. We can obtain the optimal guaranteed rate according to the first-order condition with the required second-order conditions (i.e., ∂^2^*S*(*V*,*P*_*I*_)/∂*R*^2^<0) since the asset and liability operations are non-simultaneous, as mentioned previously. Partially differentiating Eq (7) with the loan rate, the first-order condition is ∂*PP*(*V*,*P*_*I*_)/*∂R*_*C*_ = 0 for the maximum policyholder protection. We can solve the optimal loan rate based on the second-order condition ($\partial^{2}PP(V,P_{I})/\partial R_{C}^{2}<0$).

We model comparative statics about the impacts on the loan rate-setting behavior and the guaranteed rate from changes in sustainability-related parameters of the model:

$$\frac{d(R+R_{C})}{di}={\frac{\partial R}{\partial i}|}_{S}+{\frac{\partial R_{C}}{\partial i}|}_{PP}\;\forall \;i=cap,\;R_{S},\;\mathrm{and}\;\alpha$$ (8)

where

$${\frac{\partial R}{\partial i}|}_{S}=-\frac{\partial^{2}S(V,P_{I})}{\partial R\partial i}/\frac{\partial^{2}S(V,P_{I})}{\partial R^{2}},\;{\frac{\partial R_{C}}{\partial i}|}_{PP}=-\frac{\partial^{2}PP(V,P_{I})}{\partial R_{C}\partial i}/\frac{\partial^{2}PP(V,P_{I})}{\partial R_{C}^{2}}$$

The comparative statics describe the effects of the regulatory cap of the cap-and-trade mechanism, the government subsidy, and the green loan distribution rate on the two optimal rates concerning stakeholder benefit maximization. The following section describes the comparative static results.

## 4. Method and data

This study employs numerical analysis for Eq (8), which offers several advantages. The numerical analysis provides approximate solutions that align with real-life scenarios. It offers a transparent perspective on how variables and their interactions impact the comparative static results.

We define the baseline for the numerical analysis in the following:

(i) Green loan and subsidy rates: Chen et al. report that the green loan interest rate is 4.68% [23]. For the past five years (2015~2019), the average lending interest rate has been about 5.00% in Hong Kong, the interest rate can see from https://data.worldbank.org/indicator/FR.INR.LEND?locations=CN-HK. Thus, we assume that the difference between 5.00% and 4.68% is the government’s subsidy interest rate (*R*_*S*_ = 0.32%). According to the Task Force on Climate-Related Finacial Disclosures of the First Financial Holding Company, Taipei, the climate-related loans relative to total loans were about 22.10% in 2020. The Disclosures are in Chinese version. 22.10% used in the numerical analysis due to data limit may be under-estimated since the statistics are climate-related loan (i.e., climate finance, not green finance or sustainable finance). With the information above, we assume that the carbon-intensive loan demand loci faced by the insurer are (*R*_*C*_(%),*L*_*C*_) = (4.80, 321), (5.00, 320.8), (5.20, 320.4), (5.40, 319.8), (5.60, 319), (5.80, 318), and (6.00, 316.8).

(ii) Liquid-asset rate: According to the report of Gründl et al., life insurers hold relatively high liquid assets (i.e., more than 50% of the asset portfolio) due to their long-term liability-driven investment for asset-liability matching management [28]. The limitation of the liquid-asset rate falls between the green loan rate and the guaranteed rate. The statistics used by Brockman and Turtle indicate that the mean value of liquid asset interest rate is 5.81%, associated with a standard deviation of 2.07% [29]. For our research purpose of greenness, we assume *R*_*B*_(= 5.81%−2.07%) = 3.74%.

(iii) Capital ratio and investment return rates: The capital ratio for manufacturing firms is very diversified, roughly between 20% and 50%, depending on their industrial features [30]. In our model, we assume that the capital ratio is 35%. Thus we can have the value *E*_*G*_ = *αL*_*G*_×0.35/0.65 = 37.49 when *αL*_*G*_ = 69.62; similarly, *E*_*C*_ = (1−*α*)*L*_*C*_×0.35/0.65 = 169.62 when (1−*α*)*L*_*C*_ = 315. Further, the borrowing-firm investment return rate is, for simplicity, *R*_*G*_ = *R*_*C*_ = 7.00%, which meets the condition *R*_*G*_ = *R*_*C*_>*R*_*C*_.

(iv) Cap-and-trade scheme: We follow Narassimhan et al. to demonstrate a case of the cap-and-trade mechanism [31]. The regulatory cap is *cap* = 2.20%. The data is from two statistics. First, the compliance and administrative costs of the cap-and-trade scheme in the European Union in 2016 were $72,440 and $2,750 per installation, respectively. The marginal polluting cost of the carbon-intensive firm may be equal to *c*_*C*_ = 3.80% (= 2,750/72,440). Then, we can assume *c*_*G*_ = 3.80−2.20% in the spirit of the price mechanism.

(v) Life insurance policy loci: The guaranteed rate of the life insurance policy is about 3.25%~5.75% in Belgium, about 4.00% in the United States, and about 3.10%~6.00% in Japan [32]. We assume that the insurer faces an upward-sloping supply curve in the life insurance policy market. For the numerical analysis, we consider the supply loci as follows: (*R*(%),*P*) = (3.60, 623.32), (3.62, 629.32), (3.64, 634.32), (3.66, 638.32), (3.68, 641.32), (3.70, 643.32), and (3.72, 644.32). The participation level of the profit-sharing life insurance policy is *δ* = 0.85 [24].

(iv) Asset volatility: According to Trinks et al., the average value of asset volatility is about 0.2904 [33]. The model assumes that the green asset volatility *σ*_*G*_ = 0.2904−0.0500 = 0.2404, and *σ*_*C*_ = 0.2904+0.0500 = 0.3404. The difference shows that the green borrowing firms have a volatility advantage relative to the carbon-intensive borrowing firm. Thus, we have *σ* = 0.3194. To sum up, we establish Table 1 as follows:

**Table 1:** Data baseline for the numerical analysis.

variable	approximation and assumption	source
(*R*(%),*P*): policy supply	(3.60, 623.32), …, (3.72, 644.32)	[32]
*β*: leverage ratio	90.0%	[27]
*R*_*B*_: liquid-asset market rate	3.74%	[29]
*E*_*G*_: green borrowing-firm’s equity capital	37.49	[30]
*E*_*C*_: carbon-intensive borrowing firm’s equity capital	169.62	[30]
*c*_*G*_: green borrowing-firm’s marginal cost of carbon emission	0.016	[31]
*c*_*C*_: carbon-intensive borrowing firm’s marginal cost of carbon emission	0.038	[31]
*δ*: participation rate	0.85	[24]
*σ*_*G*_: green asset volatility	0.2404	[33]
*σ*_*C*_: carbon-intensive asset volatility	0.3404	[33]
*R*_*C*_: carbon-intensive loan rate	0.054	[23]
*L*_*C*_: carbon-intensive loan	319.8	[23]
*cap*: regulatory cap	0.022	[31]
*R*_*s*_: government’s subsidy rate	0.0032	[23]
*α*: loan distribution ratio	0.181	[27]

## 5. Results and discussion

Eq (8) allows us to examine three objectives for shareholders, policyholders, and stakeholders. Specifically, we will analyze the effects of the regulatory cap, green loan subsidies, and green loans on these three objectives.

### 5.1 Equity maximization for shareholders

The insurer’s objective is to set the guaranteed rate (*R*) to maximize the equity value (*S* in Eq (6)) for the shareholders. Given the first-order condition, we will present and discuss the comparative statics results, as shown in Tables 2–5.

**Table 2:** Responsiveness of the optimal *R* to *cap*.

	(*R*(%),*P*)						
*cap*(%)	(3.60, 623.32)	(3.62, 629.32)	(3.64, 634.32)	(3.66, 638.32)	(3.68, 641.32)	(3.70, 643.32)	(3.72, 644.32)
	*S*(*V*,*P*_*I*_)						
1.90	56.2568	56.3480	56.4202	56.4733	56.5076	56.5232	56.5202
2.00	56.2582	56.3494	56.4216	56.4748	56.5091	56.5247	56.5217
2.10	56.2595	56.3508	56.4230	56.4762	56.5105	56.5261	56.5231
2.20	56.2608	56.3521	56.4243	56.4775	56.5118	56.5274	56.5244
2.30	56.2619	56.3533	56.4255	56.4788	56.5131	56.5287	56.5257
2.40	56.2631	56.3545	56.4267	56.4800	56.5143	56.5299	56.5269
2.50	56.2641	56.3556	56.4278	56.4811	56.5155	56.5311	56.5281
	∂*R*/∂*cap*(%)						
1.90→2.00	-	3.1942	2.5330	1.8735	1.2143	0.5543	-
2.00→2.10	-	3.2015	2.5389	1.8778	1.2171	0.5556	-
2.10→2.20	-	3.2087	2.5447	1.8821	1.2199	0.5568	-
2.20→2.30	-	3.2160	2.5505	1.8865	1.2227	0.5581	-
2.30→2.40	-	3.2233	2.5563	1.8908	1.2256	0.5594	-
2.40→2.50	-	3.2306	2.5622	1.8951	1.2284	0.5607	-

^a^Unless otherwise indicated, *β* = 0.9, *R*_*B*_ = 3.74%, *E*_*G*_ = 37.49, *E*_*C*_ = 169.62, *c*_*G*_ = 0.016, *c*_*C*_ = 0.038, *δ* = 0.85, *σ*_*G*_ = 0.2404, *σ*_*C*_ = 0.3404, *R*_*S*_ = 0.32%, and *α* = 0.181. The shaded areas illustrate the optimal values and the comparative statics evaluated at the optimal *R*_*C*_ = 5.40%.

**Table 3:** Responsiveness of the optimal *R* to *R*_*S*_.

	(*R*(%),*P*)						
*R*_*S*_(%)	(3.60, 623.32)	(3.62, 629.32)	(3.64, 634.32)	(3.66, 638.32)	(3.68, 641.32)	(3.70, 643.32)	(3.72, 644.32)
	*S*(*V*,*P*_*I*_)						
0.26	56.2721	56.3634	56.4356	56.4888	56.5232	56.5388	56.5358
0.28	56.2683	56.3596	56.4318	56.4850	56.5194	56.5350	56.5320
0.30	56.2645	56.3559	56.4281	56.4813	56.5156	56.5312	56.5282
0.32	56.2608	56.3521	56.4243	56.4775	56.5118	56.5274	56.5244
0.34	56.2570	56.3483	56.4205	56.4737	56.5081	56.5237	56.5206
0.36	56.2532	56.3445	56.4167	56.4699	56.5043	56.5199	56.5169
0.38	56.2494	56.3407	56.4129	56.4662	56.5005	56.5161	56.5131
	∂*R*/∂*R*_*S*_(‰)						
0.26→0.28	-	9.1095	7.2243	5.3434	3.4634	1.5809	-
0.28→0.30	-	9.1107	7.2253	5.3441	3.4639	1.5811	-
0.30→0.32	-	9.1119	7.2262	5.3448	3.4643	1.5813	-
0.32→0.34	-	9.1130	7.2271	5.3455	3.4648	1.5815	-
0.34→0.36	-	9.1142	7.2281	5.3462	3.4652	1.5817	-
0.34→0.36	-	9.1154	7.2290	5.3469	3.4657	1.5819	-

^a^Unless otherwise indicated, *β* = 0.9, *R*_*B*_ = 3.74%, *E*_*G*_ = 37.49, *E*_*C*_ = 169.62, *c*_*G*_ = 0.016, *c*_*C*_ = 0.038, *δ* = 0.85, *σ*_*G*_ = 0.2404, *σ*_*C*_ = 0.3404, *cap* = 2.20%, and *α* = 0.181. The shaded areas illustrate the optimal values and the comparative statics evaluated at the optimal *R*_*C*_ = 5.40%.

**Table 4:** Soundness test: Responsiveness of the optimal *R* to *R*_*S*_ at various levels of *cap*.

	*cap*(%)						
*R*_*S*_(%)	1.90	2.00	2.10	2.20	2.30	2.40	2.50
	∂*R*/∂*R*_*S*_(‰)						
0.26→0.28	1.5699	1.5736	1.5773	1.5809	1.5846	1.5882	1.5919
0.28→0.30	1.5701	1.5738	1.5775	1.5811	1.5848	1.5884	1.5921
0.30→0.32	1.5704	1.5740	1.5777	1.5813	1.5850	1.5886	1.5923
0.32→0.34	1.5706	1.5742	1.5779	1.5815	1.5852	1.5888	1.5925
0.34→0.36	1.5708	1.5744	1.5781	1.5817	1.5854	1.5891	1.5927
0.34→0.36	1.5710	1.5746	1.5783	1.5819	1.5856	1.5893	1.5929

^a^Unless otherwise indicated, *β=* 0.9, *R*_*B*_ = 3.74%, *E*_*G*_ = 37.49, *E*_*C*_ = 169.62, *c*_*G*_ = 0.016, *c*_*C*_ = 0.038, *δ* = 0.85, *σ*_*G*_ = 0.2404, *σ*_*C*_ = 0.3404, and *α* = 0.181. All optimal values of the comparative statics at various levels of the regulatory cap are consistently at the evaluated rate of *R* = 3.70%. The shaded areas illustrate the optimal values and the comparative statics evaluated at the optimal *R*_*C*_ =5.40%.

**Table 5:** Responsiveness of the optimal *R* to *α*.

	(*R*(%),*P*)						
*α*	(3.60, 623.32)	(3.62, 629.32)	(3.64, 634.32)	(3.66, 638.32)	(3.68, 641.32)	(3.70, 643.32)	(3.72, 644.32)
	*S*(*V*,*P*_*I*_)						
0.151	56.0327	56.1230	56.1944	56.2470	56.2810	56.2964	56.2934
0.161	56.1083	56.1989	56.2706	56.3234	56.3575	56.3729	56.3699
0.171	56.1843	56.2753	56.3472	56.4002	56.4344	56.4500	56.4470
0.181	56.2608	56.3521	56.4243	56.4775	56.5118	56.5274	56.5244
0.191	56.3376	56.4293	56.5018	56.5552	56.5897	56.6053	56.6023
0.201	56.4148	56.5069	56.5797	56.6333	56.6679	56.6836	56.6806
0.211	56.4924	56.5848	56.6579	56.7117	56.7465	56.7623	56.7592
	∂*R*/∂*α*(%)						
0.151→0.161	-	3.5555	2.8272	2.0954	1.3601	0.6213	-
0.161→0.171	-	3.6372	2.8928	2.1444	1.3921	0.6360	-
0.171→0.181	-	3.7180	2.9578	2.1930	1.4238	0.6505	-
0.181→0.191	-	3.7978	3.0220	2.2409	1.4551	0.6649	-
0.191→0.201	-	3.8763	3.0852	2.2882	1.4860	0.6790	-
0.201→0.211	-	3.9534	3.1472	2.3346	1.5163	0.6929	-

^a^Unless otherwise indicated, *β* = 0.9, *R*_*B*_ = 3.74%, *E*_*G*_ = 37.49, *E*_*C*_ = 169.62, *c*_*G*_ = 0.016, *c*_*C*_ = 0.038, *δ* = 0.85, *σ*_*G*_ = 0.2404, *σ*_*C*_ = 0.3404, *cap* = 2.20%, and *R*_*S*_ = 0.32%. The shaded areas illustrate the optimal values and the comparative statics evaluated at the optimal *R*_*C*_ = 5.40%.

Governments often lower the cap in the cap-and-trade scheme to reduce carbon emissions. Table 2 demonstrates that a stringent cap leads to a decrease in the guaranteed rate. Consequently, there is a reduction in the number of life insurance policies issued by the insurer. As a result, the insurer’s provision of funds through green finance is based on a smaller fund-source base. This result suggests a reluctance to provide funds to borrowing firms. The decreased guaranteed rate corresponds to an increased interest margin, representing the spread between the green loan rate and the guaranteed rate. This increased margin benefits shareholders’ equity through effective asset-liability matching management. The strict regulatory cap on environmental improvement contributes to the increased margin. Therefore, when the government implements a strict cap policy to enhance environmental sustainability, the regulator and the insurer can achieve a win-win outcome.

As previously highlighted, our research underscores that the introduction of a stringent regulatory cap within the cap-and-trade system triggers a decline in the guaranteed rate, thus leading to an augmented interest margin, especially in an environment characterized by dragon king phenomena. This reduction in the guaranteed rate also has adverse consequences for life insurance policies, ultimately impacting the protection afforded to policyholders.

It is noteworthy that our discovery of a diminished guaranteed rate contrasts starkly with the findings presented by Chen et al., who contend that a stringent regulatory cap actually elevates the guaranteed rate [3]. However, it is essential to emphasize that our observation of deteriorating policyholder protection aligns with the conclusions put forth by Chen et al. [3].

The critical disparity between our study and Chen et al.’s research lies in the primary focus of each [3]. Our paper prioritizes the pursuit of equity maximization through active stakeholder engagement, while Chen et al.’s investigation confine itself to equity maximization solely with regard to equity holders [3]. Our findings underscore the critical significance of adopting alternative strategic approaches to asset-liability management, particularly within the context of stakeholder engagement. This emphasis aligns closely with the principles outlined in the Paris Agreement, highlighting the need for comprehensive strategies that account for diverse stakeholders and their interests.

Furthermore, Li et al. have demonstrated that a stringent regulatory cap, when pursued with the goal of equity maximization within a black swan environment, leads to an augmentation in the guaranteed rate and an improvement in policyholder protection [34]. It’s worth noting that these two outcomes differ significantly from the conclusions drawn in our study, which underscores the pivotal distinction between the maximization of equity for equity holders and the broader objective of stakeholder maximization. These divergent findings underscore the critical importance of PSI in shaping the outcomes of regulatory interventions.

The findings in Table 3 indicate that when the government increases subsidies for green loans, it leads to a higher guaranteed interest rate, thereby boosting the uptake of life insurance policies. However, this rise in the guaranteed interest rate has a downside: it reduces the insurer’s interest margin, resulting in a decrease in the equity value of the insurer. In simpler terms, increasing government subsidies for green loans encourages greater demand for these loans. Consequently, insurers raise the guaranteed rate to attract more life insurance businesses from environmentally conscious borrowers. While government subsidies do contribute to improving environmental quality, they come at a cost to taxpayers and reduce the insurer’s equity. Therefore, it is evident that there is no mutually beneficial solution for both the insurer and taxpayers when considering regulatory green subsidies.

Our findings reveal that an escalation in green loan subsidies results in an expansion of the insurer’s funding channels through the issuance of life insurance policies. This discovery suggests that the insurer exhibits a willingness to extend green loans to environmentally challenged borrowing firms, thereby contributing to their environmental improvement efforts. Significantly, our results find implicit support in the work of Du et al., where it is demonstrated that green protection subsidies have the potential to enhance the environmental performance of heavily polluting firms [35].

As previously stated, increasing the government’s green loan subsidy harms the insurer’s interest margin. To assess the viability of this impact, we conducted a comparative static analysis across different levels of the regulatory cap for a soundness test. Our findings confirm the validity of the soundness test. We demonstrate that the impact of reduced margins caused by increased subsidies becomes less pronounced when the regulatory cap becomes more stringent. Consequently, the insurer does not view green loan subsidies with stringent caps of the cap-and-trade mechanism as beneficial, as this adversely affects shareholders.

According to the findings in Table 5, it is evident that an increase in green loans leads to a higher guaranteed rate for the insurer. The increase in green loans, in comparison to carbon-intensive loans, results in higher expected repayments from green loans. This favorable scenario promotes the insurer’s green lending, which is increasingly funded by its life insurance policies at a higher guaranteed rate. As a result, both the insurer’s equity and environmental quality achieve a mutually beneficial outcome, signifying a win-win situation. These results highlight the significance of green loans in facilitating environmental improvement for intermediaries operating within the circular economy.

### 5.2 Protection maximization for policyholders

Alternatively, we can set the loan rate (*R*_*C*_) to maximize the protection value (*PP* in Eq (7)) for the policyholders. In the following, we will analyze the comparative statics results, as shown in Tables 6–8.

**Table 6:** Responsiveness of the optimal *R*_*C*_ to *cap*.

	(*R*_*C*_(%),*L*)						
*cap* (%)	(4.80, 321)	(5.00, 320.8)	(5.20, 320.4)	(5.40, 319.8)	(5.60, 319)	(5.80, 318)	(6.00, 316.8)
	*PP*(*V*,*P*_*I*_)						
1.90	536.2059	536.8753	537.3057	537.4962	537.4458	537.1534	536.6180
2.00	535.9165	536.5859	537.0166	537.2074	537.1574	536.8655	536.3308
2.10	535.6271	536.2966	536.7274	536.9186	536.8690	536.5776	536.0436
2.20	535.3378	536.0073	536.4383	536.6297	536.5806	536.2898	535.7563
2.30	535.0484	535.7180	536.1492	536.3410	536.2922	536.0019	535.4691
2.40	534.7591	535.4288	535.8601	536.0522	536.0038	535.7140	535.1819
2.50	534.4697	535.1395	535.5711	535.7634	535.7154	535.4262	534.8947
	∂*R*_*C*_/∂*cap*(%)						
1.90→2.00	-	6.4768	15.7969	25.0583	34.2645	43.4191	-
2.00→2.10	-	6.4689	15.7917	25.0558	34.2648	43.4221	-
2.10→2.20	-	6.4609	15.7864	25.0533	34.2650	43.4250	-
2.20→2.30	-	6.4529	15.7812	25.0507	34.2652	43.4279	-
2.30→2.40	-	6.4449	15.7759	25.0481	34.2653	43.4308	-
2.40→2.50	-	6.4368	15.7705	25.0455	34.2654	43.4337	-

^a^Unless otherwise indicated, *β* = 0.9, *R*_*B*_ = 3.74%, *E*_*G*_ = 37.49, *E*_*C*_ = 169.62, *c*_*G*_ = 0.016, *c*_*C*_ = 0.038, *δ* = 0.85, *σ*_*G*_ = 0.2404, *σ*_*C*_ = 0.3404, *R*_*S*_ = 0.32%, and *α* = 0.181. The shaded areas illustrate the optimal values, and the comparative statics are evaluated at the optimal *R* = 3.70%.

**Table 7:** Responsiveness of the optimal *R*_*C*_ to *R*_*S*_.

	(*R*_*C*_(%),*L*)						
*R*_*S*_(%)	(4.80, 321)	(5.00, 320.8)	(5.20, 320.4)	(5.40, 319.8)	(5.60, 319)	(5.80, 318)	(6.00, 316.8)
	*PP*(*V*,*P*_*I*_)						
0.26	535.3870	536.0566	536.4875	536.6789	536.6297	536.3388	535.8052
0.28	535.3706	536.0402	536.4711	536.6625	536.6133	536.3224	535.7889
0.30	535.3542	536.0237	536.4547	536.6461	536.5969	536.3061	535.7726
0.32	535.3378	536.0073	536.4383	536.6297	536.5806	536.2898	535.7563
0.34	535.3214	535.9909	536.4219	536.6134	536.5642	536.2734	535.7400
0.36	535.3049	535.9745	536.4055	536.5970	536.5478	536.2571	535.7237
0.38	535.2885	535.9581	536.3891	536.5806	536.5315	536.2408	535.7074
	∂*R*_*C*_/∂*R*_*S*_(%)						
0.26→0.28	-	1.8325	4.4790	7.1088	9.7230	12.3226	-
0.28→0.30	-	1.8324	4.4789	7.1088	9.7230	12.3226	-
0.30→0.32	-	1.8322	4.4788	7.1088	9.7230	12.3227	-
0.32→0.34	-	1.8321	4.4787	7.1087	9.7231	12.3227	-
0.34→0.36	-	1.8320	4.4787	7.1087	9.7231	12.3228	-
0.34→0.36	-	1.8319	4.4786	7.1086	9.7231	12.3228	-

^a^Unless otherwise indicated, *β* = 0.9, *R*_*B*_ = 3.74%, *E*_*G*_ = 37.49, *E*_*C*_ = 169.62, *c*_*G*_ = 0.016, *c*_*C*_ = 0.038, *δ* = 0.85, *σ*_*G*_ = 0.2404, *σ*_*C*_ = 0.3404, *cap* = 2.20%, and *α* = 0.181. The shaded areas illustrate the optimal values, and the comparative statics are evaluated at the optimal *R* = 3.70%.

**Table 8:** Responsiveness of the optimal *R*_*C*_ to *α*.

	(*R*_*C*_(%),*L*)						
*α*	(4.80, 321)	(5.00, 320.8)	(5.20, 320.4)	(5.40, 319.8)	(5.60, 319)	(5.80, 318)	(6.00, 316.8)
	*PP*(*V*,*P*_*I*_)						
0.151	522.0005	522.6587	523.0870	523.2842	523.2495	522.9818	522.4802
0.161	526.3389	527.0008	527.4300	527.6253	527.5859	527.3107	526.7988
0.171	530.7833	531.4490	531.8791	532.0725	532.0283	531.7454	531.2228
0.181	535.3378	536.0073	536.4383	536.6297	536.5806	536.2898	535.7563
0.191	540.0063	540.6798	541.1118	541.3011	541.2468	540.9479	540.4033
0.201	544.7934	545.4709	545.9038	546.0910	546.0315	545.7242	545.1682
0.211	549.7034	550.3850	550.8189	551.0039	550.9390	550.6232	550.0554
	∂*R*_*C*_/∂*α*						
0.151→0.161	-	0.3209	0.0785	‐0.1627	-0.4027	-0.6416	-
0.161→0.171	-	0.3238	0.0784	‐0.1658	-0.4088	-0.6507	-
0.171→0.181	-	0.3268	0.0783	‐0.1689	-0.4150	-0.6599	-
0.181→0.191	-	0.3298	0.0782	‐0.1721	-0.4213	-0.6693	-
0.191→0.201	-	0.3330	0.0782	‐0.1754	-0.4277	-0.6789	-
0.201→0.211	-	0.3362	0.0782	‐0.1787	-0.4343	-0.6887	-

^a^Unless otherwise indicated, *β* = 0.9, *R*_*B*_ = 3.74%, *E*_*G*_ = 37.49, *E*_*C*_ = 169.62, *c*_*G*_ = 0.016, *c*_*C*_ = 0.038, *δ* = 0.85, *σ*_*G*_ = 0.2404, *σ*_*C*_ = 0.3404, *cap* = 2.20%, and *R*_*S*_ = 0.32%. The shaded areas illustrate the optimal value, and the comparative statics are evaluated at the optimal *R* = 3.70%.

When the regulatory caps are lowered within the cap-and-trade mechanism, it increases lending at a lower loan rate, primarily aimed at maximizing policyholder protection, as illustrated in Table 6. The stringent caps serve as a stimulus for borrowing firms to enhance their green operations, promoting sustainable development. The surge in lending activity creates a higher demand for funds in the life insurance business, facilitating efficient asset-liability matching management and ultimately leading to increased policyholder protection. In light of these findings, we can argue that no ideal solution simultaneously improves environmental quality, preserves insurer margin, and protects policyholders when the government implements strict caps within the cap-and-trade scheme.

Increasing the government’s green loan subsidies makes it possible to raise the loan interest rate and maximize the protection of policyholders, as demonstrated in Table 7. This increase in the loan rate leads to a reduction in lending and an expansion of the insurer’s spread, primarily due to the imperfectly competitive nature of the loan market faced by the insurer. It is important to note that green loan subsidies result in environmental improvements but come at taxpayers’ expense. From a stakeholder perspective, policyholders emerge as the winners, as the insurer prioritizes maximizing their protection. Additionally, the insurer itself benefits from increased margins in this scenario. However, it is essential to acknowledge that both winners, policyholders, and the insurer comes at a cost to taxpayers when the government subsidizes green development.

As depicted in Table 8, it becomes apparent that an escalation in green loans results in a decrease in loan rate settings when the insurer aims to prioritize the protection of policyholders. Even at a constant subsidy level, the increased volume of green loans leads to reduced green loan repayments from borrowing firms. This reduction subsequently diminishes the insurer’s interest margin. Consequently, from the perspective of insurance protection, no mutually beneficial solution satisfies the insurer’s shareholders and policyholders. It is worth noting that while the increase in green lending boosts the insurer’s margin in terms of maximizing shareholders’ equity (as shown in Table 5), it concurrently diminishes the margin concerning maximizing policyholder protection (as demonstrated in Table 8). In this case of green lending, differing objectives set by the insurer may create conflicts of interest among stakeholders.

A crucial managerial implication derived from the findings is that insurers must cautiously balance their objectives when implementing green lending practices. Although increasing green loans can augment shareholder equity, it may inadvertently compromise policyholder protection. Insurers must delicately strike a balance between maximizing shareholder returns and guaranteeing sufficient protection for policyholders. This argument emphasizes the significance of developing a comprehensive understanding of stakeholder interests and employing effective risk management strategies to mitigate potential conflicts and optimize overall stakeholder value in green lending.

### 5.3 Benefit maximization for stakeholders

Considering benefits for stakeholders is essential for sustainable development. For simplicity, the research only models the stakeholders, including shareholders and policyholders. The results derived from our model may not extend to situations where stakeholders include not only shareholders and policyholders, for example in addition to borrowing firms, taxpayers and regulators. Table 9 presents the comparative static results from the viewpoint of stakeholders.

**Table 9:** The total effects of *cap*, *R*_*S*_, and *α* on *R*+*R*_*C*_: According to Eq (8).

*cap*(%)	∂*R*/∂*cap*(%)	*R*_*S*_(%)	∂*R*/∂*R*_*S*_(‰)	*α*	∂*R*/∂*α*(%)
1.90→2.00	0.5543	0.26→0.28	1.5809	0.151→0.161	0.6213
2.00→2.10	0.5556	0.28→0.30	1.5811	0.161→0.171	0.6360
2.10→2.20	0.5568	0.30→0.32	1.5813	0.171→0.181	0.6505
2.20→2.30	0.5581	0.32→0.34	1.5815	0.181→0.191	0.6649
2.30→2.40	0.5594	0.34→0.36	1.5817	0.191→0.201	0.6790
2.40→2.50	0.5607	0.34→0.36	1.5819	0.201→0.211	0.6929
*cap*(%)	∂*R*_*C*_/∂*cap*(%)	*R*_*S*_(%)	∂*R*_*C*_/∂*R*_*S*_(%)	*α*	∂*R*_*C*_/∂*α*
1.90→2.00	25.0583	0.26→0.28	7.1088	0.151→0.161	-0.1627
2.00→2.10	25.0558	0.28→0.30	7.1088	0.161→0.171	-0.1658
2.10→2.20	25.0533	0.30→0.32	7.1088	0.171→0.181	-0.1689
2.20→2.30	25.0507	0.32→0.34	7.1087	0.181→0.191	-0.1721
2.30→2.40	25.0481	0.34→0.36	7.1087	0.191→0.201	-0.1754
2.40→2.50	25.0455	0.34→0.36	7.1086	0.201→0.211	-0.1787
*cap*(%)	*d*(*R*+*R*_*C*_)/*dcap* (%)	*R*_*S*_(%)	*d*(*R*+*R*_*C*_)/*dR*_*S*_ (%)	*α*	*d*(*R*+*R*_*C*_)/*dα*
1.90→2.00	25.6126	0.26→0.28	7.26689	0.151→0.161	-0.1565
2.00→2.10	25.6114	0.28→0.30	7.26691	0.161→0.171	-0.1594
2.10→2.20	25.6101	0.30→0.32	7.26693	0.171→0.181	-0.1624
2.20→2.30	25.6088	0.32→0.34	7.26685	0.181→0.191	-0.1655
2.30→2.40	25.6075	0.34→0.36	7.26687	0.191→0.201	-0.1686
2.40→2.50	25.6062	0.34→0.36	7.26679	0.201→0.211	-0.1718

^a^The comparative statics are evaluated at the optima *R* = 3.70% and *R*_*C*_ = 5.40%.

In the first panel, Tables 2, 3, and 5 illustrate the effects of the regulatory cap, green subsidies, and green loans on the optimal guaranteed rate, focusing on equity maximization. The second panel, summarized from Tables 6–8, provides insights into these effects from the perspective of policyholder protection. The third panel presents comparative static results, demonstrating that reducing the regulatory cap leads to a simultaneous decrease in guaranteed and loan rates. This outcome aligns with the insurer’s objective of maximizing benefits for stakeholders, including shareholders and policyholders. It becomes evident that the impact of a strict cap policy is more substantial when considering the interests of stakeholders as a whole rather than solely focusing on either equity maximization for shareholders or policyholder protection. From the perspective of carbon emission regulations, the pursuit of stakeholder benefit maximization proves highly efficient.

In their studies, Chen et al. delve into the cap-and-trade system while incorporating green finance from insurers, explicitly concentrating on choices related to green technology [3]. Li et al. thoroughly examine the cap-and-trade scheme, coupled with incentives in the form of green loan subsidies, with a keen focus on the environmental impacts of borrowing firms [4]. Huang and Lin take a different approach by developing a contingent claim model to analyze insurer-led green lending within the context of the cap-and-trade mechanism, particularly in a time of conflict [13]. Collectively, these works share a common theme of exploring carbon trading within the cap-and-trade framework and its intersection with insurer-driven green finance.

These studies collectively shed light on the regulatory cap’s impact on the guaranteed rate of life insurance policies and its implications for maximizing equity. However, our primary finding stands in difference: reducing these caps has the effect of diminishing both guaranteed and loan rates, thereby aligning with the objective of maximizing stakeholder benefits. This finding, in turn, makes a unique contribution to the field of insurer-driven green finance, particularly when considering stakeholder engagement: a facet that Kujala et al.’s comprehensive literature review remains conspicuously silent on [8].

The findings presented in the second term of the third panel indicate that increasing green loan subsidies results in higher guaranteed and loan rates. It can be summarized that the impact of a green loan subsidy policy is more significant when considering stakeholders’ interests as a whole rather than focusing solely on individual interests. Examining the results on the right-hand side of the third panel, we observe that increasing green loans leads to a higher guaranteed rate but a lower loan rate. When prioritizing stakeholders’ interests, the magnitude of the impact of green loans becomes less critical than maximizing policyholders’ protection.

Policy implications, focusing on sustainable production, include the need for policymakers to adopt a stakeholder-oriented approach in insurance policies that promote sustainable production practices while striking a balance between equity maximization for shareholders and policyholder protection. Encouraging the implementation of green loan subsidies can incentivize insurers to adopt sustainable production practices, benefiting stakeholders and supporting the transition to greener and more sustainable insurance operations. Holistic stakeholder engagement should prioritize the interests of shareholders, policyholders, borrowing firms, and regulators in driving sustainable production efforts. Carbon emission regulations should align with stakeholder benefit maximization and sustainable production goals, ensuring policy coherence and avoiding conflicting outcomes between regulatory caps and green loans.

## 6. Conclusions

The pivotal conclusion of this paper underscores the critical role of sustainable insurance in advancing both green finance and policyholder protection within the framework of UNEP FI PSI. Our study employs a capped call option model to meticulously assess insurer equity and liabilities, with a strong emphasis on delivering tangible stakeholder benefits. It is evident from our analysis that stricter regulatory caps exert downward pressure on insurer margins, while the presence of government subsidies for green loans introduces potential risks to policyholder protection. In the context of these regulatory caps and government subsidies, the pursuit of a mutually beneficial outcome that balances the interests of both shareholders and policyholders in sustainable insurance emerges as a complex and challenging endeavor. This conclusion highlights the multifaceted nature of the sustainable insurance landscape and the imperative need for a nuanced approach to ensure the sustainable and equitable growth of this vital industry.

This research carries profound implications for understanding the intricate dynamics of insurer stakeholder management, particularly in the context of striving for sustainable production to achieve mutually beneficial results. An immediate and tangible application of our findings emerges. For instance, when we delve into the realm of government subsidies, we discern a complex interplay. Governments aspire to foster environmental improvement, albeit at the expense of taxpayers. Insurers, armed with adept stakeholder management strategies, stand to reap substantial benefits. Policyholders, in turn, experience heightened protection while the position and interests of borrowing firms remain shrouded in ambiguity. Yet, it is vital to acknowledge the formidable challenge that looms large: the task of forging sustainable insurance solutions that can harmoniously accommodate the diverse needs and aspirations of all stakeholders. This implication underscores the intricate web of interests and responsibilities inherent in the pursuit of sustainable insurance and underscores the critical importance of strategic and inclusive approaches.

It is imperative to acknowledge the inherent limitations of our research when considering its practical applications. In the real world, the stakeholder landscape is shaped by a multitude of factors that may not perfectly align with the precise expectations delineated in our examination of stakeholder management within the PSI framework. Specifically, the intricacies of strategic decisions within investment and insurance markets, often influenced by asymmetric information, can introduce substantial complexities. It is vital to recognize that these complexities extend beyond the boundaries of our study, and we have not delved into addressing these intricacies within this particular context. Future research endeavors should explore these intricate dimensions, providing a deeper understanding of how strategic considerations and asymmetric information may influence stakeholder dynamics within sustainable insurance. These uncharted territories present fertile ground for expanding our knowledge and refining our approaches in this critical field.

## Supporting information

S1 Table Simulation. (XLSX)

S2 Table Computations of Tables 2–9. (DOCX)
