# Supplementary material for: Balancing equity and policyholder protection: Assessing insurer’s interests in green lending under cap-and-trade regulations
Source: PLoS One. 2023 Nov 6;18(11):e0293975. doi: 10.1371/journal.pone.0293975 (PMC10627471; doi:10.1371/journal.pone.0293975)
Supplement: S2 Table — (DOCX) [file pone.0293975.s002.docx]

| **Table 2** | | | | | | | |
| --- | --- | --- | --- | --- | --- | --- | --- |
| Responsiveness of the optimal $R$ to $cap$.^a^ | | | | | | | |
|  | $(R(\%), P)$ | | | | | | |
| $cap$(%) | (3.60, 623.32) | (3.62, 629.32) | (3.64, 634.32) | (3.66, 638.32) | (3.68, 641.32) | (3.70, 643.32) | (3.72, 644.32) |
|  | $S(V,P_{I})$ | | | | | | |
| 1.90 | 56.2567920524 | 56.3480324790 | 56.4201573829 | 56.4733099894 | 56.5076218875 | 56.5232133625 | 56.5201936774 |
| 2.00 | 56.2581764742 | 56.3494474300 | 56.4215963628 | 56.4747666181 | 56.5090898823 | 56.5246865151 | 56.5216658313 |
| 2.10 | 56.2594977209 | 56.3507992860 | 56.4229723109 | 56.4761602616 | 56.5104949221 | 56.5260967264 | 56.5230750414 |
| 2.20 | 56.2607555609 | 56.3520878153 | 56.4242849957 | 56.4774906886 | 56.5118367755 | 56.5274437650 | 56.5244210761 |
| 2.30 | 56.2619497622 | 56.3533127861 | 56.4255341855 | 56.4787576674 | 56.5131152108 | 56.5287273993 | 56.5257037039 |
| 2.40 | 56.2630800927 | 56.3544739663 | 56.4267196482 | 56.4799609659 | 56.5143299962 | 56.5299473974 | 56.5269226927 |
| 2.50 | 56.2641463196 | 56.3555711234 | 56.4278411513 | 56.4811003517 | 56.5154808992 | 56.5311035269 | 56.5280778103 |
|  | $\partial^{2}S(V,P_{I})/\partial R^{2}$ | | | | | | |
| 1.90 | - | -47.7888067500 | -47.4307435000 | -47.1017710000 | -46.8010577500 | -46.5279002500 | - |
| 2.00 | - | -47.8050575000 | -47.4466937500 | -47.1174777500 | -46.8165785000 | -46.5432915000 | - |
| 2.10 | - | -47.8213505000 | -47.4626855000 | -47.1332255000 | -46.8321405000 | -46.5587232500 | - |
| 2.20 | - | -47.8376850000 | -47.4787187500 | -47.1490150000 | -46.8477435000 | -46.5741960000 | - |
| 2.30 | - | -47.8540612500 | -47.4947937500 | -47.1648462500 | -46.8633872500 | -46.5897097500 | - |
| 2.40 | - | -47.8704792500 | -47.5109105000 | -47.1807185000 | -46.8790727500 | -46.6052647500 | - |
| 2.50 | - | -47.8869397500 | -47.5270687500 | -47.1966322500 | -46.8947995000 | -46.6208607500 | - |
|  | $\partial^{2}S(V,P_{I})/\partial R\partial cap$ | | | | | | |
| 1.90→2.00 | 1.5264600002 | 1.2014450000 | 0.8824399998 | 0.5683050002 | 0.2578899998 | -0.0499350001 | - |
| 2.00→2.10 | 1.5304649999 | 1.2046049999 | 0.8847700002 | 0.5698149998 | 0.2585750000 | -0.0500599999 | - |
| 2.10→2.20 | 1.5344650002 | 1.2077749997 | 0.8871100000 | 0.5713200000 | 0.2592600001 | -0.0501950002 | - |
| 2.20→2.30 | 1.5384750000 | 1.2109500002 | 0.8894499999 | 0.5728250002 | 0.2599500000 | -0.0503250000 | - |
| 2.30→2.40 | 1.5424849998 | 1.2141250000 | 0.8917900001 | 0.5743449996 | 0.2606350002 | -0.0504650000 | - |
| 2.40→2.50 | 1.5465099999 | 1.2173000002 | 0.8941350000 | 0.5758600000 | 0.2613249997 | -0.0505949998 | - |
|  | $\partial R/\partial cap$ | | | | | | |
| 1.90→2.00 | - | 0.0319417894 | 0.0253305116 | 0.0187347520 | 0.0121429948 | 0.0055426959 | - |
| 2.00→2.10 | - | 0.0320147089 | 0.0253885973 | 0.0187779576 | 0.0121712226 | 0.0055555804 | - |
| 2.10→2.20 | - | 0.0320874460 | 0.0254468323 | 0.0188213302 | 0.0121993143 | 0.0055684517 | - |
| 2.20→2.30 | - | 0.0321603146 | 0.0255051112 | 0.0188646571 | 0.0122273765 | 0.0055814168 | - |
| 2.30→2.40 | - | 0.0322331054 | 0.0255633282 | 0.0189079382 | 0.0122557295 | 0.0055942611 | - |
| 2.40→2.50 | - | 0.0323061315 | 0.0256214833 | 0.0189512799 | 0.0122839460 | 0.0056071991 | - |
| ^a^ Unless otherwise indicated, $\beta=$ 0.9, $R_{B}=$ 3.74%, $E_{G}=$ 37.49, $E_{C}=$ 169.62, $c_{G}=$ 0.016, $c_{C}=$ 0.038, $\delta=$ 0.85, $\sigma_{G}=$ 0.2404, $\sigma_{C}=$ 0.3404, $R_{S}=$ 0.32%, and $\alpha=$ 0.181. The shaded areas illustrate the optimal values, and the comparative statics evaluated at the optimal $R_{C}=$ 5.40%. | | | | | | | |

| **Table 3** | | | | | | | |
| --- | --- | --- | --- | --- | --- | --- | --- |
| Responsiveness of the optimal $R$ to $R_{S}$.^a^ | | | | | | | |
|  | $(R(\%), P)$ | | | | | | |
| $R_{S}$(%) | (3.60, 623.32) | (3.62, 629.32) | (3.64, 634.32) | (3.66, 638.32) | (3.68, 641.32) | (3.70, 643.32) | (3.72, 644.32) |
|  | $S(V,P_{I})$ | | | | | | |
| 0.26 | 56.2721011971 | 56.3634282217 | 56.4356212857 | 56.4888239551 | 56.5231680947 | 56.5387742006 | 56.5357516828 |
| 0.28 | 56.2683193533 | 56.3596481208 | 56.4318425568 | 56.4850462339 | 56.5193910225 | 56.5349974229 | 56.5319748481 |
| 0.30 | 56.2645374745 | 56.3558679854 | 56.4280637935 | 56.4812684784 | 56.5156139161 | 56.5312206110 | 56.5281979792 |
| 0.32 | 56.2607555609 | 56.3520878153 | 56.4242849957 | 56.4774906886 | 56.5118367755 | 56.5274437650 | 56.5244210761 |
| 0.34 | 56.2569736123 | 56.3483076105 | 56.4205061635 | 56.4737128645 | 56.5080596006 | 56.5236668848 | 56.5206441389 |
| 0.36 | 56.2531916289 | 56.3445273711 | 56.4167272968 | 56.4699350061 | 56.5042823916 | 56.5198899704 | 56.5168671674 |
| 0.38 | 56.2494096105 | 56.3407470970 | 56.4129483957 | 56.4661571134 | 56.5005051483 | 56.5161130219 | 56.5130901618 |
|  | $\partial^{2}S(V,P_{I})/\partial R^{2}$ | | | | | | |
| 0.26 | - | -47.8349012452 | -47.4759866970 | -47.1463243984 | -46.8450843218 | -46.5715593015 | - |
| 0.28 | - | -47.8358289564 | -47.4768973071 | -47.1472211736 | -46.8459705051 | -46.5724380999 | - |
| 0.30 | - | -47.8367568014 | -47.4778080510 | -47.1481180828 | -46.8468568212 | -46.5733170297 | - |
| 0.32 | - | -47.8376850000 | -47.4787187500 | -47.1490150000 | -46.8477435000 | -46.5741960000 | - |
| 0.34 | - | -47.8386128962 | -47.4796299416 | -47.1499123005 | -46.8486298502 | -46.5750752868 | - |
| 0.36 | - | -47.8395411457 | -47.4805410882 | -47.1508096089 | -46.8495165645 | -46.5759546128 | - |
| 0.38 | - | -47.8404695302 | -47.4814523685 | -47.1517070506 | -46.8504034112 | -46.5768340719 | - |
|  | $\partial^{2}S(V,P_{I})/\partial R\partial R_{S}$ | | | | | | |
| 0.26→0.28 | 0.4357532433 | 0.3429821192 | 0.2519211009 | 0.1622435875 | 0.0736252517 | -0.0142545815 | - |
| 0.28→0.30 | 0.4358177108 | 0.3430332072 | 0.2519588236 | 0.1622678951 | 0.0736362864 | -0.0142566918 | - |
| 0.30→0.32 | 0.4358889747 | 0.3430691180 | 0.2519992162 | 0.1623075008 | 0.0736396224 | -0.0142574130 | - |
| 0.32→0.34 | 0.4359400840 | 0.3431504680 | 0.2520313078 | 0.1623012622 | 0.0736662393 | -0.0142624366 | - |
| 0.34→0.36 | 0.4360113550 | 0.3431864002 | 0.2520717359 | 0.1623408963 | 0.0736694688 | -0.0142631365 | - |
| 0.36→0.38 | 0.4360758794 | 0.3432374314 | 0.2521094089 | 0.1623652324 | 0.0736805603 | -0.0142653462 | - |
|  | $\partial R/\partial R_{S}$ | | | | | | |
| 0.26→0.28 | - | 0.0091095253 | 0.0072243284 | 0.0053433879 | 0.0034634069 | 0.0015809059 | - |
| 0.28→0.30 | - | 0.0091106963 | 0.0072252659 | 0.0053440864 | 0.0034638603 | 0.0015811130 | - |
| 0.30→0.32 | - | 0.0091120093 | 0.0072258837 | 0.0053448415 | 0.0034646401 | 0.0015811548 | - |
| 0.32→0.34 | - | 0.0091129009 | 0.0072274585 | 0.0053454204 | 0.0034644414 | 0.0015816964 | - |
| 0.34→0.36 | - | 0.0091142140 | 0.0072280766 | 0.0053461761 | 0.0034652219 | 0.0015817359 | - |
| 0.36→0.38 | - | 0.0091153859 | 0.0072290126 | 0.0053468734 | 0.0034656757 | 0.0015819442 | - |
| ^a^ Unless otherwise indicated, $\beta=$ 0.9, $R_{B}=$ 3.74%, $E_{G}=$ 37.49, $E_{C}=$ 169.62, $c_{G}=$ 0.016, $c_{C}=$ 0.038, $\delta=$ 0.85, $\sigma_{G}=$ 0.2404, $\sigma_{C}=$ 0.3404, $cap=$ 2.20%, and $\alpha=$ 0.181. The shaded areas illustrate the optimal values, and the comparative statics evaluated at the optimal $R_{C}=$ 5.40%. | | | | | | | |

| **Table 4-1** | | | | | | | |
| --- | --- | --- | --- | --- | --- | --- | --- |
| Responsiveness of the optimal $R$ to $R_{S}$ at $cap=$ 1.90%.^a^ | | | | | | | |
|  | $(R(\%), P)$ | | | | | | |
| $R_{S}$(%) | (3.60, 623.32) | (3.62, 629.32) | (3.64, 634.32) | (3.66, 638.32) | (3.68, 641.32) | (3.70, 643.32) | (3.72, 644.32) |
|  | $S(V,P_{I})$ | | | | | | |
| 0.26 | 56.2681325686 | 56.3593678062 | 56.4314886261 | 56.4846382329 | 56.5189481992 | 56.5345387976 | 56.5315192822 |
| 0.28 | 56.2643524311 | 56.3555893981 | 56.4277115792 | 56.4808621857 | 56.5151727959 | 56.5307636864 | 56.5277441144 |
| 0.30 | 56.2605722590 | 56.3518109557 | 56.4239344981 | 56.4770861045 | 56.5113973586 | 56.5269885414 | 56.5239689128 |
| 0.32 | 56.2567920524 | 56.3480324790 | 56.4201573829 | 56.4733099894 | 56.5076218875 | 56.5232133625 | 56.5201936774 |
| 0.34 | 56.2530118111 | 56.3442539679 | 56.4163802336 | 56.4695338402 | 56.5038463825 | 56.5194381498 | 56.5164184081 |
| 0.34 | 56.2492315353 | 56.3404754225 | 56.4126030502 | 56.4657576571 | 56.5000708436 | 56.5156629032 | 56.5126431049 |
| 0.36 | 56.2454512248 | 56.3366968427 | 56.4088258326 | 56.4619814400 | 56.4962952708 | 56.5118876228 | 56.5088677679 |
|  | $\partial^{2}S(V,P_{I})/\partial R^{2}$ | | | | | | |
| 0.26 | - | -47.7860442705 | -47.4280329077 | -47.0991010748 | -46.7984199879 | -46.5252843956 | - |
| 0.28 | - | -47.7869648716 | -47.4289364419 | -47.0999908121 | -46.7992991708 | -46.5261562312 | - |
| 0.30 | - | -47.7878856059 | -47.4298401112 | -47.1008806815 | -46.8001784859 | -46.5270281978 | - |
| 0.32 | - | -47.7888064758 | -47.4307439129 | -47.1017706847 | -46.8010579322 | -46.5279002967 | - |
| 0.34 | - | -47.7897274793 | -47.4316478479 | -47.1026608205 | -46.8019375123 | -46.5287725266 | - |
| 0.34 | - | -47.7906486167 | -47.4325519178 | -47.1035510892 | -46.8028172233 | -46.5296448882 | - |
| 0.36 | - | -47.7915698891 | -47.4334561208 | -47.1044414907 | -46.8036970666 | -46.5305173814 | - |
|  | $\partial^{2}S(V,P_{I})/\partial R\partial R_{S}$ | | | | | | |
| 0.26→0.28 | 0.4323475125 | 0.3402873929 | 0.2499339757 | 0.1609602478 | 0.0730419529 | -0.0141416052 | - |
| 0.28→0.30 | 0.4324117668 | 0.3403383388 | 0.2499714142 | 0.1609844702 | 0.0730529663 | -0.0141436942 | - |
| 0.30→0.32 | 0.4324761562 | 0.3403891711 | 0.2500089948 | 0.1610086713 | 0.0730640366 | -0.0141458614 | - |
| 0.32→0.34 | 0.4325403964 | 0.3404400459 | 0.2500465470 | 0.1610329718 | 0.0730749647 | -0.0141480214 | - |
| 0.34→0.36 | 0.4326047431 | 0.3404909990 | 0.2500840139 | 0.1610571445 | 0.0730860421 | -0.0141501175 | - |
| 0.34→0.36 | 0.4326690828 | 0.3405418454 | 0.2501215448 | 0.1610813953 | 0.0730970626 | -0.0141522563 | - |
|  | $\partial R/\partial R_{S}$ | | | | | | |
| 0.26→0.28 | - | 0.0090475686 | 0.0071748157 | 0.0053065551 | 0.0034394377 | 0.0015699410 | - |
| 0.28→0.30 | - | 0.0090487389 | 0.0071757531 | 0.0053072497 | 0.0034398906 | 0.0015701483 | - |
| 0.30→0.32 | - | 0.0090499119 | 0.0071766881 | 0.0053079474 | 0.0034403431 | 0.0015703568 | - |
| 0.32→0.34 | - | 0.0090510818 | 0.0071776240 | 0.0053086443 | 0.0034407977 | 0.0015705623 | - |
| 0.34→0.36 | - | 0.0090522538 | 0.0071785615 | 0.0053093394 | 0.0034412495 | 0.0015707709 | - |
| 0.34→0.36 | - | 0.0090534256 | 0.0071794966 | 0.0053100358 | 0.0034417030 | 0.0015709783 | - |
| ^a^ Unless otherwise indicated, $\beta=$ 0.9, $R_{B}=$ 3.74%, $E_{G}=$ 37.49, $E_{C}=$ 169.62, $c_{G}=$ 0.016, $c_{C}=$ 0.038, $\delta=$ 0.85, $\sigma_{G}=$ 0.2404, $\sigma_{C}=$ 0.3404, and $\alpha=$ 0.181. The shaded areas illustrate the optimal values, and the comparative statics evaluated at the optimal $R_{C}=$ 5.40%. | | | | | | | |

| **Table 4-2** | | | | | | | |
| --- | --- | --- | --- | --- | --- | --- | --- |
| Responsiveness of the optimal $R$ to $R_{S}$ at $cap=$ 2.00%.^a^ | | | | | | | |
|  | $(R(\%), P)$ | | | | | | |
| $R_{S}$(%) | (3.60, 623.32) | (3.62, 629.32) | (3.64, 634.32) | (3.66, 638.32) | (3.68, 641.32) | (3.70, 643.32) | (3.72, 644.32) |
|  | $S(V,P_{I})$ | | | | | | |
| 0.26 | 56.2695186863 | 56.3607844395 | 56.4329292775 | 56.4860965252 | 56.5204178524 | 56.5360136063 | 56.5329930927 |
| 0.28 | 56.2657379836 | 56.3570054708 | 56.4291516735 | 56.4823199235 | 56.5166418964 | 56.5322379431 | 56.5292173729 |
| 0.30 | 56.2619572462 | 56.3532264676 | 56.4253740353 | 56.4785432878 | 56.5128659063 | 56.5284622461 | 56.5254416191 |
| 0.32 | 56.2581764742 | 56.3494474300 | 56.4215963628 | 56.4747666181 | 56.5090898823 | 56.5246865151 | 56.5216658313 |
| 0.34 | 56.2543956675 | 56.3456683580 | 56.4178186561 | 56.4709899142 | 56.5053138243 | 56.5209107501 | 56.5178900096 |
| 0.34 | 56.2506148260 | 56.3418892515 | 56.4140409152 | 56.4672131762 | 56.5015377322 | 56.5171349512 | 56.5141141540 |
| 0.36 | 56.2468339498 | 56.3381101105 | 56.4102631400 | 56.4634364042 | 56.4977616062 | 56.5133591184 | 56.5103382644 |
|  | $\partial^{2}S(V,P_{I})/\partial R^{2}$ | | | | | | |
| 0.26 | - | -47.8022881696 | -47.4439759494 | -47.1148008457 | -46.8139336506 | -46.5406684710 | - |
| 0.28 | - | -47.8032111373 | -47.4448818405 | -47.1156929257 | -46.8148151646 | -46.5415426243 | - |
| 0.30 | - | -47.8041342397 | -47.4457878645 | -47.1165851403 | -46.8156968089 | -46.5424169106 | - |
| 0.32 | - | -47.8050574768 | -47.4466940224 | -47.1174774865 | -46.8165785878 | -46.5432913270 | - |
| 0.34 | - | -47.8059808483 | -47.4476003149 | -47.1183699654 | -46.8174604984 | -46.5441658753 | - |
| 0.34 | - | -47.8069043538 | -47.4485067411 | -47.1192625773 | -46.8183425419 | -46.5450405551 | - |
| 0.36 | - | -47.8078279946 | -47.4494133002 | -47.1201553233 | -46.8192247170 | -46.5459153667 | - |
|  | $\partial^{2}S(V,P_{I})/\partial R\partial R_{S}$ | | | | | | |
| 0.26→0.28 | 0.4334814037 | 0.3411846379 | 0.2505955266 | 0.1613875327 | 0.0732361372 | -0.0141792000 | - |
| 0.28→0.30 | 0.4335457859 | 0.3412355412 | 0.2506331427 | 0.1614116840 | 0.0732472500 | -0.0141813814 | - |
| 0.30→0.32 | 0.4336101753 | 0.3412864729 | 0.2506706807 | 0.1614360556 | 0.0732581711 | -0.0141834633 | - |
| 0.32→0.34 | 0.4336746073 | 0.3413374543 | 0.2507082044 | 0.1614603136 | 0.0732692484 | -0.0141855807 | - |
| 0.34→0.36 | 0.4337389541 | 0.3413884002 | 0.2507457779 | 0.1614845857 | 0.0732802405 | -0.0141877408 | - |
| 0.34→0.36 | 0.4338033861 | 0.3414393106 | 0.2507834083 | 0.1615088081 | 0.0732912895 | -0.0141898724 | - |
|  | $\partial R/\partial R_{S}$ | | | | | | |
| 0.26→0.28 | - | 0.0090682145 | 0.0071913163 | 0.0053188281 | 0.0034474252 | 0.0015735944 | - |
| 0.28→0.30 | - | 0.0090693863 | 0.0071922519 | 0.0053195258 | 0.0034478761 | 0.0015738037 | - |
| 0.30→0.32 | - | 0.0090705581 | 0.0071931880 | 0.0053202217 | 0.0034483318 | 0.0015740087 | - |
| 0.32→0.34 | - | 0.0090717307 | 0.0071941251 | 0.0053209174 | 0.0034487850 | 0.0015742172 | - |
| 0.34→0.36 | - | 0.0090729015 | 0.0071950615 | 0.0053216140 | 0.0034492385 | 0.0015744238 | - |
| 0.34→0.36 | - | 0.0090740740 | 0.0071959970 | 0.0053223118 | 0.0034496909 | 0.0015746316 | - |
| ^a^ Unless otherwise indicated, $\beta=$ 0.9, $R_{B}=$ 3.74%, $E_{G}=$ 37.49, $E_{C}=$ 169.62, $c_{G}=$ 0.016, $c_{C}=$ 0.038, $\delta=$ 0.85, $\sigma_{G}=$ 0.2404, $\sigma_{C}=$ 0.3404, and $\alpha=$ 0.181. The shaded areas illustrate the optimal values, and the comparative statics evaluated at the optimal $R_{C}=$ 5.40%. | | | | | | | |

| **Table 4-3** | | | | | | | |
| --- | --- | --- | --- | --- | --- | --- | --- |
| Responsiveness of the optimal $R$ to $R_{S}$ at $cap$=2.10%.^a^ | | | | | | | |
|  | $(R(\%), P)$ | | | | | | |
| $R_{S}$(%) | (3.60, 623.32) | (3.62, 629.32) | (3.64, 634.32) | (3.66, 638.32) | (3.68, 641.32) | (3.70, 643.32) | (3.72, 644.32) |
|  | $S(V,P_{I})$ | | | | | | |
| 0.26 | 56.2708416396 | 56.3621379885 | 56.4343069079 | 56.4874918430 | 56.5218245614 | 56.5374254844 | 56.5344039700 |
| 0.28 | 56.2670603682 | 56.3583584555 | 56.4305287432 | 56.4837146834 | 56.5180480490 | 56.5336492657 | 56.5306276945 |
| 0.30 | 56.2632790620 | 56.3545788880 | 56.4267505442 | 56.4799374896 | 56.5142715026 | 56.5298730131 | 56.5268513850 |
| 0.32 | 56.2594977209 | 56.3507992860 | 56.4229723109 | 56.4761602616 | 56.5104949221 | 56.5260967264 | 56.5230750414 |
| 0.34 | 56.2557163451 | 56.3470196494 | 56.4191940433 | 56.4723829994 | 56.5067183074 | 56.5223204056 | 56.5192986637 |
| 0.34 | 56.2519349344 | 56.3432399782 | 56.4154157413 | 56.4686057031 | 56.5029416587 | 56.5185440508 | 56.5155222520 |
| 0.36 | 56.2481534889 | 56.3394602725 | 56.4116374049 | 56.4648283725 | 56.4991649759 | 56.5147676619 | 56.5117458062 |
|  | $\partial^{2}S(V,P_{I})/\partial R^{2}$ | | | | | | |
| 0.26 | - | -47.8185738127 | -47.4599605309 | -47.1305419374 | -46.8294884121 | -46.5560934229 | - |
| 0.28 | - | -47.8194991508 | -47.4608687804 | -47.1314363634 | -46.8303722592 | -46.5569698977 | - |
| 0.30 | - | -47.8204246235 | -47.4617771629 | -47.1323309237 | -46.8312562384 | -46.5578465035 | - |
| 0.32 | - | -47.8213502312 | -47.4626856794 | -47.1332256166 | -46.8321403504 | -46.5587232426 | - |
| 0.34 | - | -47.8222759727 | -47.4635943308 | -47.1341204420 | -46.8330245955 | -46.5596001116 | - |
| 0.34 | - | -47.8232018495 | -47.4645031149 | -47.1350154017 | -46.8339089720 | -46.5604771142 | - |
| 0.36 | - | -47.8241278604 | -47.4654120344 | -47.1359104939 | -46.8347934817 | -46.5613542470 | - |
|  | $\partial^{2}S(V,P_{I})/\partial R\partial R_{S}$ | | | | | | |
| 0.26→0.28 | 0.4346166733 | 0.3420828634 | 0.2512579087 | 0.1618153149 | 0.0734305985 | -0.0142168801 | - |
| 0.28→0.30 | 0.4346810840 | 0.3421338235 | 0.2512955817 | 0.1618395444 | 0.0734416261 | -0.0142189478 | - |
| 0.30→0.32 | 0.4347456084 | 0.3421848334 | 0.2513331765 | 0.1618638876 | 0.0734526893 | -0.0142212215 | - |
| 0.32→0.34 | 0.4348100262 | 0.3422358716 | 0.2513707287 | 0.1618881882 | 0.0734636743 | -0.0142232324 | - |
| 0.34→0.36 | 0.4348745009 | 0.3422868247 | 0.2514084159 | 0.1619124461 | 0.0734748014 | -0.0142254564 | - |
| 0.34→0.36 | 0.4349389968 | 0.3423379127 | 0.2514459680 | 0.1619367538 | 0.0734857863 | -0.0142274956 | - |
|  | $\partial R/\partial R_{S}$ | | | | | | |
| 0.26→0.28 | - | 0.0090888673 | 0.0072078202 | 0.0053311059 | 0.0034554150 | 0.0015772500 | - |
| 0.28→0.30 | - | 0.0090900384 | 0.0072087560 | 0.0053318040 | 0.0034558671 | 0.0015774572 | - |
| 0.30→0.32 | - | 0.0090912118 | 0.0072096928 | 0.0053325005 | 0.0034563217 | 0.0015776651 | - |
| 0.32→0.34 | - | 0.0090923829 | 0.0072106301 | 0.0053331960 | 0.0034567753 | 0.0015778713 | - |
| 0.34→0.36 | - | 0.0090935551 | 0.0072115656 | 0.0053338943 | 0.0034572280 | 0.0015780806 | - |
| 0.34→0.36 | - | 0.0090947277 | 0.0072125039 | 0.0053345897 | 0.0034576818 | 0.0015782868 | - |
| ^a^ Unless otherwise indicated, $\beta=$ 0.9, $R_{B}=$ 3.74%, $E_{G}=$ 37.49, $E_{C}=$ 169.62, $c_{G}=$ 0.016, $c_{C}=$ 0.038, $\delta=$ 0.85, $\sigma_{G}=$ 0.2404, $\sigma_{C}=$ 0.3404, and $\alpha=$ 0.181. The shaded areas illustrate the optimal values, and the comparative statics evaluated at the optimal $R_{C}=$ 5.40%. | | | | | | | |

| **Table 4-4** | | | | | | | |
| --- | --- | --- | --- | --- | --- | --- | --- |
| Responsiveness of the optimal $R$ to $R_{S}$ at $cap$=2.20%.^a^ | | | | | | | |
|  | $(R(\%), P)$ | | | | | | |
| $R_{S}$(%) | (3.60, 623.32) | (3.62, 629.32) | (3.64, 634.32) | (3.66, 638.32) | (3.68, 641.32) | (3.70, 643.32) | (3.72, 644.32) |
|  | $S(V,P_{I})$ | | | | | | |
| 0.26 | 56.2721011970964 | 56.3634282216566 | 56.4356212857189 | 56.4888239551023 | 56.5231680947264 | 56.5387742006217 | 56.5357516827965 |
| 0.28 | 56.2683193532731 | 56.3596481208463 | 56.4318425568370 | 56.4850462339049 | 56.5193910225033 | 56.5349974228996 | 56.5319748480561 |
| 0.30 | 56.2645374745448 | 56.3558679853889 | 56.4280637935124 | 56.4812684784156 | 56.5156139160856 | 56.5312206110271 | 56.5281979791567 |
| 0.32 | 56.2607555609052 | 56.3520878152782 | 56.4242849957388 | 56.4774906886276 | 56.5118367754666 | 56.5274437649977 | 56.5244210760918 |
| 0.34 | 56.2569736123476 | 56.3483076105079 | 56.4205061635098 | 56.4737128645350 | 56.5080596006401 | 56.5236668848050 | 56.5206441388553 |
| 0.34 | 56.2531916288661 | 56.3445273710718 | 56.4167272968193 | 56.4699350061315 | 56.5042823916001 | 56.5198899704429 | 56.5168671674406 |
| 0.36 | 56.2494096104542 | 56.3407470969634 | 56.4129483956606 | 56.4661571134104 | 56.5005051483400 | 56.5161130219051 | 56.5130901618414 |
|  | $\partial^{2}S(V,P_{I})/\partial R^{2}$ | | | | | | |
| 0.26 | - | -47.8349012451673 | -47.4759866969521 | -47.1463243984260 | -46.8450843217738 | -46.5715593015403 | - |
| 0.28 | - | -47.8358289564085 | -47.4768973071349 | -47.1472211735602 | -46.8459705051316 | -46.5724380998722 | - |
| 0.30 | - | -47.8367568014448 | -47.4778080509708 | -47.1481180828448 | -46.8468568212188 | -46.5733170296545 | - |
| 0.32 | - | -47.8376847811290 | -47.4787189297388 | -47.1490151242904 | -46.8477432697512 | -46.5741960923793 | - |
| 0.34 | - | -47.8386128961716 | -47.4796299415914 | -47.1499123004548 | -46.8486298502313 | -46.5750752867677 | - |
| 0.34 | - | -47.8395411457200 | -47.4805410882340 | -47.1508096088513 | -46.8495165645066 | -46.5759546128197 | - |
| 0.36 | - | -47.8404695302004 | -47.4814523684586 | -47.1517070506167 | -46.8504034112271 | -46.5768340718853 | - |
|  | $\partial^{2}S(V,P_{I})/\partial R\partial R_{S}$ | | | | | | |
| 0.26→0.28 | 0.4357532432664 | 0.3429821191503 | 0.2519211008689 | 0.1622435874538 | 0.0736252516731 | -0.0142545815152 | - |
| 0.28→0.30 | 0.4358177108088 | 0.3430332071730 | 0.2519588235828 | 0.1622678951207 | 0.0736362864018 | -0.0142566918271 | - |
| 0.30→0.32 | 0.4358822423001 | 0.3430842738794 | 0.2519963970826 | 0.1622922525257 | 0.0736473992902 | -0.0142588731933 | - |
| 0.32→0.34 | 0.4359468164239 | 0.3431353121641 | 0.2520341269019 | 0.1623165104547 | 0.0736584624406 | -0.0142609763998 | - |
| 0.34→0.36 | 0.4360113550206 | 0.3431864001868 | 0.2520717359289 | 0.1623408962814 | 0.0736694687475 | -0.0142631364497 | - |
| 0.34→0.36 | 0.4360758794064 | 0.3432374313661 | 0.2521094089047 | 0.1623652323701 | 0.0736805603196 | -0.0142653462376 | - |
|  | $\partial R/\partial R_{S}$ | | | | | | |
| 0.26→0.28 | - | 0.0091095253031 | 0.0072243284029 | 0.0053433879329 | 0.0034634068826 | 0.0015809058743 | - |
| 0.28→0.30 | - | 0.0091106963194 | 0.0072252659005 | 0.0053440864024 | 0.0034638602503 | 0.0015811129803 | - |
| 0.30→0.32 | - | 0.0091118686016 | 0.0072262028928 | 0.0053447816653 | 0.0034643146529 | 0.0015813217522 | - |
| 0.32→0.34 | - | 0.0091130417038 | 0.0072271392299 | 0.0053454802022 | 0.0034647669050 | 0.0015815294438 | - |
| 0.34→0.36 | - | 0.0091142139921 | 0.0072280765585 | 0.0053461761354 | 0.0034652218603 | 0.0015817359026 | - |
| 0.34→0.36 | - | 0.0091153859122 | 0.0072290126334 | 0.0053468733834 | 0.0034656757268 | 0.0015819441798 | - |
| ^a^ Unless otherwise indicated, $\beta=$ 0.9, $R_{B}=$ 3.74%, $E_{G}=$ 37.49, $E_{C}=$ 169.62, $c_{G}=$ 0.016, $c_{C}=$ 0.038, $\delta=$ 0.85, $\sigma_{G}=$ 0.2404, $\sigma_{C}=$ 0.3404, and $\alpha=$ 0.181. The shaded areas illustrate the optimal values, and the comparative statics evaluated at the optimal $R_{C}=$ 5.40%. | | | | | | | |

| **Table 4-5** | | | | | | | |
| --- | --- | --- | --- | --- | --- | --- | --- |
| Responsiveness of the optimal $R$ to $R_{S}$ at $cap$=2.30%.^a^ | | | | | | | |
|  | $(R(\%), P)$ | | | | | | |
| $R_{S}$(%) | (3.60, 623.32) | (3.62, 629.32) | (3.64, 634.32) | (3.66, 638.32) | (3.68, 641.32) | (3.70, 643.32) | (3.72, 644.32) |
|  | $S(V,P_{I})$ | | | | | | |
| 0.26 | 56.2732971268 | 56.3646549072 | 56.4368721794 | 56.4900926298 | 56.5244482209 | 56.5400595234 | 56.5370359995 |
| 0.28 | 56.2695147070 | 56.3608742349 | 56.4330928827 | 56.4863143434 | 56.5206705852 | 56.5362821830 | 56.5332586019 |
| 0.30 | 56.2657322521 | 56.3570935279 | 56.4293135514 | 56.4825360226 | 56.5168929152 | 56.5325048083 | 56.5294811700 |
| 0.32 | 56.2619497622 | 56.3533127861 | 56.4255341855 | 56.4787576674 | 56.5131152108 | 56.5287273993 | 56.5257037039 |
| 0.34 | 56.2581672373 | 56.3495320095 | 56.4217547851 | 56.4749792778 | 56.5093374722 | 56.5249499561 | 56.5219262034 |
| 0.34 | 56.2543846774 | 56.3457511982 | 56.4179753501 | 56.4712008537 | 56.5055596992 | 56.5211724786 | 56.5181486687 |
| 0.36 | 56.2506020824 | 56.3419703521 | 56.4141958805 | 56.4674223952 | 56.5017818919 | 56.5173949668 | 56.5143710997 |
|  | $\partial^{2}S(V,P_{I})/\partial R^{2}$ | | | | | | |
| 0.26 | - | -47.8512705094 | -47.4920544943 | -47.1621482772 | -46.8607214293 | -46.5870661561 | - |
| 0.28 | - | -47.8522005941 | -47.4929674689 | -47.1630474038 | -46.8616099511 | -46.5879472821 | - |
| 0.30 | - | -47.8531308146 | -47.4938805773 | -47.1639466634 | -46.8624986071 | -46.5888285382 | - |
| 0.32 | - | -47.8540611707 | -47.4947938183 | -47.1648460583 | -46.8633873942 | -46.5897099281 | - |
| 0.34 | - | -47.8549916607 | -47.4957071952 | -47.1657455849 | -46.8642763154 | -46.5905914493 | - |
| 0.34 | - | -47.8559222853 | -47.4966207061 | -47.1666452459 | -46.8651653689 | -46.5914731025 | - |
| 0.36 | - | -47.8568530453 | -47.4975343504 | -47.1675450405 | -46.8660545558 | -46.5923548885 | - |
|  | $\partial^{2}S(V,P_{I})/\partial R\partial R_{S}$ | | | | | | |
| 0.26→0.28 | 0.4368909927 | 0.3438825260 | 0.2525850675 | 0.1626724071 | 0.0738202246 | -0.0142923824 | - |
| 0.28→0.30 | 0.4369556450 | 0.3439335927 | 0.2526227476 | 0.1626967858 | 0.0738311883 | -0.0142944145 | - |
| 0.30→0.32 | 0.4370202618 | 0.3439846594 | 0.2526605627 | 0.1627210722 | 0.0738423651 | -0.0142966243 | - |
| 0.32→0.34 | 0.4370848572 | 0.3440358540 | 0.2526981646 | 0.1627455006 | 0.0738533785 | -0.0142987489 | - |
| 0.34→0.36 | 0.4371494455 | 0.3440869847 | 0.2527358944 | 0.1627697941 | 0.0738644417 | -0.0143008734 | - |
| 0.34→0.36 | 0.4372140765 | 0.3441380798 | 0.2527736456 | 0.1627941870 | 0.0738754977 | -0.0143031045 | - |
|  | $\partial R/\partial R_{S}$ | | | | | | |
| 0.26→0.28 | - | 0.0091301858 | 0.0072408433 | 0.0053556735 | 0.0034714021 | 0.0015845648 | - |
| 0.28→0.30 | - | 0.0091313595 | 0.0072417794 | 0.0053563703 | 0.0034718565 | 0.0015847702 | - |
| 0.30→0.32 | - | 0.0091325323 | 0.0072427154 | 0.0053570700 | 0.0034723089 | 0.0015849801 | - |
| 0.32→0.34 | - | 0.0091337046 | 0.0072436540 | 0.0053577651 | 0.0034727643 | 0.0015851865 | - |
| 0.34→0.36 | - | 0.0091348766 | 0.0072445912 | 0.0053584628 | 0.0034732168 | 0.0015853939 | - |
| 0.34→0.36 | - | 0.0091360495 | 0.0072455277 | 0.0053591610 | 0.0034736715 | 0.0015856012 | - |
| ^a^ Unless otherwise indicated, $\beta=$ 0.9, $R_{B}=$ 3.74%, $E_{G}=$ 37.49, $E_{C}=$ 169.62, $c_{G}=$ 0.016, $c_{C}=$ 0.038, $\delta=$ 0.85, $\sigma_{G}=$ 0.2404, $\sigma_{C}=$ 0.3404, and $\alpha=$ 0.181. The shaded areas illustrate the optimal values, and the comparative statics evaluated at the optimal $R_{C}=$ 5.40%. | | | | | | | |

| **Table 4-6** | | | | | | | |
| --- | --- | --- | --- | --- | --- | --- | --- |
| Responsiveness of the optimal $R$ to $R_{S}$ at $cap$=2.40%.^a^ | | | | | | | |
|  | $(R(\%), P)$ | | | | | | |
| $R_{S}$(%) | (3.60, 623.32) | (3.62, 629.32) | (3.64, 634.32) | (3.66, 638.32) | (3.68, 641.32) | (3.70, 643.32) | (3.72, 644.32) |
|  | $S(V,P_{I})$ | | | | | | |
| 0.26 | 56.2744291965 | 56.3658178130 | 56.4380593569 | 56.4912976351 | 56.5256647079 | 56.5412812208 | 56.5382566881 |
| 0.28 | 56.2706461970 | 56.3620365657 | 56.4342794886 | 56.4875187799 | 56.5218865051 | 56.5375033141 | 56.5344787240 |
| 0.30 | 56.2668631624 | 56.3582552834 | 56.4304995857 | 56.4837398901 | 56.5181082679 | 56.5337253729 | 56.5307007255 |
| 0.32 | 56.2630800927 | 56.3544739663 | 56.4267196482 | 56.4799609659 | 56.5143299962 | 56.5299473974 | 56.5269226927 |
| 0.34 | 56.2592969878 | 56.3506926143 | 56.4229396759 | 56.4761820071 | 56.5105516901 | 56.5261693875 | 56.5231446254 |
| 0.34 | 56.2555138477 | 56.3469112274 | 56.4191596690 | 56.4724030138 | 56.5067733496 | 56.5223913432 | 56.5193665238 |
| 0.36 | 56.2517306726 | 56.3431298057 | 56.4153796274 | 56.4686239859 | 56.5029949746 | 56.5186132645 | 56.5155883878 |
|  | $\partial^{2}S(V,P_{I})/\partial R^{2}$ | | | | | | |
| 0.26 | - | -47.8676816471 | -47.5081639693 | -47.1780136203 | -46.8763997827 | -46.6026140376 | - |
| 0.28 | - | -47.8686141104 | -47.5090793089 | -47.1789151015 | -46.8772906466 | -46.6034974924 | - |
| 0.30 | - | -47.8695467081 | -47.5099947830 | -47.1798167166 | -46.8781816439 | -46.6043810787 | - |
| 0.32 | - | -47.8704794410 | -47.5109103914 | -47.1807184653 | -46.8790727739 | -46.6052647981 | - |
| 0.34 | - | -47.8714123086 | -47.5118261348 | -47.1816203461 | -46.8799640380 | -46.6061486495 | - |
| 0.34 | - | -47.8723453109 | -47.5127420123 | -47.1825223625 | -46.8808554341 | -46.6070326322 | - |
| 0.36 | - | -47.8732784494 | -47.5136580236 | -47.1834245123 | -46.8817469628 | -46.6079167480 | - |
|  | $\partial^{2}S(V,P_{I})/\partial R\partial R_{S}$ | | | | | | |
| 0.26→0.28 | 0.4380301419 | 0.3447838139 | 0.2532498513 | 0.1631017312 | 0.0740153396 | -0.0143301477 | - |
| 0.28→0.30 | 0.4380948013 | 0.3448350299 | 0.2532876238 | 0.1631261100 | 0.0740263815 | -0.0143322438 | - |
| 0.30→0.32 | 0.4381594820 | 0.3448861960 | 0.2533253536 | 0.1631504887 | 0.0740374944 | -0.0143344465 | - |
| 0.32→0.34 | 0.4382241414 | 0.3449373764 | 0.2533630408 | 0.1631749598 | 0.0740485504 | -0.0143365853 | - |
| 0.34→0.36 | 0.4382888434 | 0.3449886208 | 0.2534008701 | 0.1631992248 | 0.0740596064 | -0.0143386671 | - |
| 0.34→0.36 | 0.4383535810 | 0.3450397301 | 0.2534385999 | 0.1632236177 | 0.0740707549 | -0.0143408272 | - |
|  | $\partial R/\partial R_{S}$ | | | | | | |
| 0.26→0.28 | - | 0.0091508535 | 0.0072573593 | 0.0053679634 | 0.0034793997 | 0.0015882229 | - |
| 0.28→0.30 | - | 0.0091520260 | 0.0072582975 | 0.0053686615 | 0.0034798536 | 0.0015884297 | - |
| 0.30→0.32 | - | 0.0091531989 | 0.0072592346 | 0.0053693586 | 0.0034803075 | 0.0015886381 | - |
| 0.32→0.34 | - | 0.0091543713 | 0.0072601719 | 0.0053700547 | 0.0034807634 | 0.0015888452 | - |
| 0.34→0.36 | - | 0.0091555445 | 0.0072611105 | 0.0053707539 | 0.0034812148 | 0.0015890523 | - |
| 0.34→0.36 | - | 0.0091567183 | 0.0072620463 | 0.0053714509 | 0.0034816689 | 0.0015892613 | - |
| ^a^ Unless otherwise indicated, $\beta=$ 0.9, $R_{B}=$ 3.74%, $E_{G}=$ 37.49, $E_{C}=$ 169.62, $c_{G}=$ 0.016, $c_{C}=$ 0.038, $\delta=$ 0.85, $\sigma_{G}=$ 0.2404, $\sigma_{C}=$ 0.3404, and $\alpha=$ 0.181. The shaded areas illustrate the optimal values, and the comparative statics evaluated at the optimal $R_{C}=$ 5.40%. | | | | | | | |

| **Table 4-7** | | | | | | | |
| --- | --- | --- | --- | --- | --- | --- | --- |
| Responsiveness of the optimal $R$ to $R_{S}$ at $cap$=2.50%.^a^ | | | | | | | |
|  | $(R(\%), P)$ | | | | | | |
| $R_{S}$(%) | (3.60, 623.32) | (3.62, 629.32) | (3.64, 634.32) | (3.66, 638.32) | (3.68, 641.32) | (3.70, 643.32) | (3.72, 644.32) |
|  | $S(V,P_{I})$ | | | | | | |
| 0.26 | 56.2754971738 | 56.3669167067 | 56.4391825858 | 56.4924387388 | 56.5268173236 | 56.5424390606 | 56.5394135165 |
| 0.28 | 56.2717135910 | 56.3631348806 | 56.4354021424 | 56.4886593111 | 56.5230385500 | 56.5386605838 | 56.5356349822 |
| 0.30 | 56.2679299729 | 56.3593530195 | 56.4316216642 | 56.4848798487 | 56.5192597418 | 56.5348820726 | 56.5318564135 |
| 0.32 | 56.2641463196 | 56.3555711234 | 56.4278411513 | 56.4811003517 | 56.5154808992 | 56.5311035269 | 56.5280778103 |
| 0.34 | 56.2603626311 | 56.3517891923 | 56.4240606035 | 56.4773208201 | 56.5117020220 | 56.5273249466 | 56.5242991725 |
| 0.34 | 56.2565789073 | 56.3480072262 | 56.4202800210 | 56.4735412538 | 56.5079231102 | 56.5235463319 | 56.5205205003 |
| 0.36 | 56.2527951482 | 56.3442252251 | 56.4164994037 | 56.4697616529 | 56.5041441640 | 56.5197676827 | 56.5167417936 |
|  | $\partial^{2}S(V,P_{I})/\partial R^{2}$ | | | | | | |
| 0.26 | - | -47.8841347005 | -47.5243151652 | -47.1939204766 | -46.8921194313 | -46.6182029937 | - |
| 0.28 | - | -47.8850695440 | -47.5252328736 | -47.1948243139 | -46.8930126409 | -46.6190887808 | - |
| 0.30 | - | -47.8860045210 | -47.5261507170 | -47.1957282861 | -46.8939059824 | -46.6199746992 | - |
| 0.32 | - | -47.8869396339 | -47.5270686944 | -47.1966323916 | -46.8947994575 | -46.6208607508 | - |
| 0.34 | - | -47.8878748821 | -47.5279868067 | -47.1975366305 | -46.8956930662 | -46.6217469341 | - |
| 0.34 | - | -47.8888102646 | -47.5289050534 | -47.1984410039 | -46.8965868081 | -46.6226332502 | - |
| 0.36 | - | -47.8897457833 | -47.5298234340 | -47.1993455115 | -46.8974806819 | -46.6235196994 | - |
|  | $\partial^{2}S(V,P_{I})/\partial R\partial R_{S}$ | | | | | | |
| 0.26→0.28 | 0.4391705559 | 0.3456862103 | 0.2539153741 | 0.1635316451 | 0.0742106891 | -0.0143680197 | - |
| 0.28→0.30 | 0.4392352082 | 0.3457375044 | 0.2539531607 | 0.1635559457 | 0.0742217949 | -0.0143700518 | - |
| 0.30→0.32 | 0.4392999884 | 0.3457886990 | 0.2539909616 | 0.1635804026 | 0.0742328936 | -0.0143722616 | - |
| 0.32→0.34 | 0.4393647615 | 0.3458399433 | 0.2540287127 | 0.1636048239 | 0.0742439497 | -0.0143743790 | - |
| 0.34→0.36 | 0.4394294777 | 0.3458912232 | 0.2540665562 | 0.1636292239 | 0.0742550341 | -0.0143765746 | - |
| 0.34→0.36 | 0.4394942934 | 0.3459424249 | 0.2541043642 | 0.1636535956 | 0.0742662110 | -0.0143787133 | - |
|  | $\partial R/\partial R_{S}$ | | | | | | |
| 0.26→0.28 | - | 0.0091715254 | 0.0072738809 | 0.0053802560 | 0.0034874014 | 0.0015918822 | - |
| 0.28→0.30 | - | 0.0091726965 | 0.0072748198 | 0.0053809536 | 0.0034878532 | 0.0015920902 | - |
| 0.30→0.32 | - | 0.0091738702 | 0.0072757565 | 0.0053816515 | 0.0034883083 | 0.0015922980 | - |
| 0.32→0.34 | - | 0.0091750437 | 0.0072766942 | 0.0053823483 | 0.0034887626 | 0.0015925049 | - |
| 0.34→0.36 | - | 0.0091762159 | 0.0072776325 | 0.0053830470 | 0.0034892165 | 0.0015927124 | - |
| 0.34→0.36 | - | 0.0091773901 | 0.0072785692 | 0.0053837449 | 0.0034896696 | 0.0015929218 | - |
| ^a^ Unless otherwise indicated, $\beta=$ 0.9, $R_{B}=$ 3.74%, $E_{G}=$ 37.49, $E_{C}=$ 169.62, $c_{G}=$ 0.016, $c_{C}=$ 0.038, $\delta=$ 0.85, $\sigma_{G}=$ 0.2404, $\sigma_{C}=$ 0.3404, and $\alpha=$ 0.181. The shaded areas illustrate the optimal values, and the comparative statics evaluated at the optimal $R_{C}=$ 5.40%. | | | | | | | |

| **Table 5** | | | | | | | |
| --- | --- | --- | --- | --- | --- | --- | --- |
| Responsiveness of the optimal $R$ to $\alpha$.^a^ | | | | | | | |
|  | $(R(\%), P)$ | | | | | | |
| $\alpha$ | (3.60, 623.32) | (3.62, 629.32) | (3.64, 634.32) | (3.66, 638.32) | (3.68, 641.32) | (3.70, 643.32) | (3.72, 644.32) |
|  | $S(V,P_{I})$ | | | | | | |
| 0.151 | 56.0327326561 | 56.1230289724 | 56.1944083745 | 56.2470120579 | 56.2809698273 | 56.2964004429 | 56.2934119094 |
| 0.161 | 56.1082964069 | 56.1989290154 | 56.2705738299 | 56.3233728635 | 56.3574566255 | 56.3729444630 | 56.3699448485 |
| 0.171 | 56.1843127347 | 56.2752906530 | 56.3472080573 | 56.4002077581 | 56.4344209573 | 56.4499675851 | 56.4469565857 |
| 0.181 | 56.2607555609 | 56.3520878153 | 56.4242849957 | 56.4774906886 | 56.5118367755 | 56.5274437650 | 56.5244210761 |
| 0.191 | 56.3375971483 | 56.4292927577 | 56.5017768974 | 56.5551939062 | 56.5896763310 | 56.6053452535 | 56.6023105707 |
| 0.201 | 56.4148080609 | 56.5068760206 | 56.5796542859 | 56.6332879234 | 56.6679101299 | 56.6836425540 | 56.6805955732 |
| 0.211 | 56.4923571257 | 56.5848063893 | 56.6578859161 | 56.7117414738 | 56.7465068926 | 56.7623043810 | 56.7592447995 |
|  | $\partial^{2}S(V,P_{I})/\partial R^{2}$ | | | | | | |
| 0.151 | - | -47.2922852694 | -46.9392968442 | -46.6147848687 | -46.3178846847 | -46.0478726745 | - |
| 0.161 | - | -47.4694848451 | -47.1144523510 | -46.7881791084 | -46.4898112411 | -46.2186299833 | - |
| 0.171 | - | -47.6512851063 | -47.2942588148 | -46.9662539840 | -46.6664282673 | -46.3940683424 | - |
| 0.181 | - | -47.8376847811 | -47.4787189297 | -47.1490151243 | -46.8477432698 | -46.5741960924 | - |
| 0.191 | - | -48.0286744156 | -47.6678272110 | -47.3364599889 | -47.0337556196 | -46.7590134678 | - |
| 0.201 | - | -48.2242359219 | -47.8615695267 | -47.5285774015 | -47.2244560697 | -46.9485121256 | - |
| 0.211 | - | -48.4243421133 | -48.0599226327 | -47.7253470701 | -47.4198262752 | -47.1426746597 | - |
|  | $\partial^{2}S(V,P_{I})/\partial R\partial\alpha$ | | | | | | |
| 0.151→0.161 | 1.6814612715 | 1.3270621201 | 0.9767511065 | 0.6299626273 | 0.2861095146 | -0.0554051030 |  |
| 0.161→0.171 | 1.7265491995 | 1.3629486770 | 1.0033357495 | 0.6471859983 | 0.2939519459 | -0.0569247722 |  |
| 0.171→0.181 | 1.7716801854 | 1.3988808358 | 1.0299606059 | 0.6644383252 | 0.3018083203 | -0.0584471798 |  |
| 0.181→0.191 | 1.8167753166 | 1.4347960476 | 1.0565794851 | 0.6816897559 | 0.3096650562 | -0.0599696946 |  |
| 0.191→0.201 | 1.8617511509 | 1.4706281384 | 1.0831435070 | 0.6989086818 | 0.3175077815 | -0.0614895340 |  |
| 0.201→0.211 | 1.9065196550 | 1.5063072722 | 1.1096010601 | 0.7160617230 | 0.3253213120 | -0.0630037563 |  |
|  | $\partial R/\partial\alpha$ | | | | | | |
| 0.151→0.161 | - | 0.0355546631 | 0.0282718790 | 0.0209536762 | 0.0136008505 | 0.0062133058 | - |
| 0.161→0.171 | - | 0.0363717703 | 0.0289284627 | 0.0214442145 | 0.0139210287 | 0.0063600316 | - |
| 0.171→0.181 | - | 0.0371801134 | 0.0295782378 | 0.0219298010 | 0.0142380368 | 0.0065053213 | - |
| 0.181→0.191 | - | 0.0379779106 | 0.0302197717 | 0.0224093649 | 0.0145511760 | 0.0066488546 | - |
| 0.191→0.201 | - | 0.0387633257 | 0.0308515874 | 0.0228818020 | 0.0148597252 | 0.0067903011 | - |
| 0.201→0.211 | - | 0.0395344710 | 0.0314721662 | 0.0233459767 | 0.0151629427 | 0.0069293210 | - |
| ^a^ Unless otherwise indicated, $\beta=$ 0.9, $R_{B}=$ 3.74%, $E_{G}=$ 37.49, $E_{C}=$ 169.62, $c_{G}=$ 0.016, $c_{C}=$ 0.038, $\delta=$ 0.85, $\sigma_{G}=$ 0.2404, $\sigma_{C}=$ 0.3404, $cap=$ 2.20%, and $R_{S}=$ 0.32%. The shaded areas illustrate the optimal values, and the comparative statics evaluated at the optimal $R_{C}=$ 5.40%. | | | | | | | |

| **Table 6** | | | | | | | |
| --- | --- | --- | --- | --- | --- | --- | --- |
| Responsiveness of the optimal $R_{C}$ to $cap$.^a^ | | | | | | | |
|  | $(R_{C}(\%), L)$ | | | | | | |
| $cap$(%) | (4.80, 321) | (5.00, 320.8) | (5.20, 320.4) | (5.40, 319.8) | (5.60, 319) | (5.80, 318) | (6.00, 316.8) |
|  | $PP(V,P_{I})$ | | | | | | |
| 1.90 | 536.2059320933 | 536.8752606003 | 537.3056953616 | 537.4962066230 | 537.4457740394 | 537.1533863679 | 536.6180411643 |
| 2.00 | 535.9165337270 | 536.5859395971 | 537.0165638605 | 537.2073770038 | 537.1573589439 | 536.8654987225 | 536.3307942020 |
| 2.10 | 535.6271463463 | 536.2966294485 | 536.7274430629 | 536.9185579170 | 536.8689541904 | 536.5776212093 | 536.0435571436 |
| 2.20 | 535.3377699865 | 536.0073301896 | 536.4383330034 | 536.6297493969 | 536.5805598127 | 536.2897538620 | 535.7563300222 |
| 2.30 | 535.0484046828 | 535.7180418552 | 536.1492337167 | 536.3409514780 | 536.2921758449 | 536.0018967141 | 535.4691128710 |
| 2.40 | 534.7590504706 | 535.4287644806 | 535.8601452377 | 536.0521641946 | 536.0038023210 | 535.7140497992 | 535.1819057230 |
| 2.50 | 534.4697073852 | 535.1394981007 | 535.5710676010 | 535.7633875813 | 535.7154392752 | 535.4262131510 | 534.8947086116 |
|  | $\partial^{2}PP(V,P_{I})/\partial R_{C}^{2}$ | | | | | | |
| 1.90 | - | -5.9723436425 | -5.9980874975 | -6.0235961250 | -6.0488771975 | -6.0739383025 | - |
| 2.00 | - | -5.9695401675 | -5.9952780025 | -6.0207800800 | -6.0460540375 | -6.0711074775 | - |
| 2.10 | - | -5.9667371950 | -5.9924690075 | -6.0179645175 | -6.0432313625 | -6.0682771150 | - |
| 2.20 | - | -5.9639347325 | -5.9896605075 | -6.0151494425 | -6.0404091625 | -6.0654472275 | - |
| 2.30 | - | -5.9611327725 | -5.9868525050 | -6.0123348600 | -6.0375874425 | -6.0626178075 | - |
| 2.40 | - | -5.9583313225 | -5.9840450050 | -6.0095207625 | -6.0347662050 | -6.0597888600 | - |
| 2.50 | - | -5.9555303800 | -5.9812380000 | -6.0067071600 | -6.0319454525 | -6.0569603800 | - |
|  | $\partial^{2}PP(V,P_{I})/\partial R_{C}\partial cap$ | | | | | | |
| 1.90→2.00 | 0.3868155005 | 0.9475104997 | 1.5094094999 | 2.0726184999 | 2.6372504999 | 3.2034154998 | - |
| 2.00→2.10 | 0.3861604995 | 0.9467550001 | 1.5085540002 | 2.0716665000 | 2.6362015001 | 3.2022739998 | - |
| 2.10→2.20 | 0.3855045003 | 0.9459969999 | 1.5076969999 | 2.0707120001 | 2.6351520000 | 3.2011295002 | - |
| 2.20→2.30 | 0.3848465002 | 0.9452385001 | 1.5068389996 | 2.0697555004 | 2.6340994998 | 3.1999834999 | - |
| 2.30→2.40 | 0.3841879999 | 0.9444780000 | 1.5059780003 | 2.0687974995 | 2.6330450004 | 3.1988345000 | - |
| 2.40→2.50 | 0.3835275004 | 0.9437159997 | 1.5051169999 | 2.0678375000 | 2.6319879998 | 3.1976840000 | - |
|  | $\partial R_{C}/\partial cap$ | | | | | | |
| 1.90→2.00 | - | 0.0647677903 | 0.1579687692 | 0.2505827862 | 0.3426451608 | 0.4341911901 | - |
| 2.00→2.10 | - | 0.0646884833 | 0.1579167805 | 0.2505578978 | 0.3426476983 | 0.4342208584 | - |
| 2.10→2.20 | - | 0.0646089291 | 0.1578643125 | 0.2505327167 | 0.3426497971 | 0.4342504388 | - |
| 2.20→2.30 | - | 0.0645289591 | 0.1578116988 | 0.2505073255 | 0.3426515398 | 0.4342795182 | - |
| 2.30→2.40 | - | 0.0644488245 | 0.1577586886 | 0.2504813912 | 0.3426530082 | 0.4343082615 | - |
| 2.40→2.50 | - | 0.0643682735 | 0.1577053647 | 0.2504554122 | 0.3426541194 | 0.4343365851 | - |
| ^a^ Unless otherwise indicated, $\beta=$ 0.9, $R_{B}=$ 3.74%, $E_{G}=$ 37.49, $E_{C}=$ 169.62, $c_{G}=$ 0.016, $c_{C}=$ 0.038, $\delta=$ 0.85, $\sigma_{G}=$ 0.2404, $\sigma_{C}=$ 0.3404, $R_{S}=$ 0.32%, and $\alpha=$ 0.181. The shaded areas illustrate the optimal values, and the comparative statics are evaluated at the optimal $R=$ 3.70  %. | | | | | | | |

| **Table 7** | | | | | | | |
| --- | --- | --- | --- | --- | --- | --- | --- |
| Responsiveness of the optimal $R_{C}$ to $R_{S}$.^a^ | | | | | | | |
|  | $(R_{C}(\%), L)$ | | | | | | |
| $R_{S}$(%) | (4.80, 321) | (5.00, 320.8) | (5.20, 320.4) | (5.40, 319.8) | (5.60, 319) | (5.80, 318) | (6.00, 316.8) |
|  | $PP(V,P_{I})$ | | | | | | |
| 0.26 | 535.3870288875 | 536.0565759762 | 536.4875465956 | 536.6789116738 | 536.6296516085 | 536.3387559623 | 535.8052231603 |
| 0.28 | 535.3706092183 | 536.0401606788 | 536.4711420302 | 536.6625242141 | 536.6132876431 | 536.3224218960 | 535.7889254155 |
| 0.30 | 535.3541895846 | 536.0237454166 | 536.4547374995 | 536.6461367884 | 536.5969237111 | 536.3060878626 | 535.7726277028 |
| 0.32 | 535.3377699865 | 536.0073301896 | 536.4383330034 | 536.6297493969 | 536.5805598127 | 536.2897538620 | 535.7563300222 |
| 0.34 | 535.3213504240 | 535.9909149977 | 536.4219285420 | 536.6133620396 | 536.5641959478 | 536.2734198943 | 535.7400323737 |
| 0.36 | 535.3049308970 | 535.9744998410 | 536.4055241153 | 536.5969747163 | 536.5478321165 | 536.2570859594 | 535.7237347573 |
| 0.38 | 535.2885114058 | 535.9580847195 | 536.3891197233 | 536.5805874272 | 536.5314683186 | 536.2407520573 | 535.7074371730 |
|  | $\partial^{2}PP(V,P_{I})/\partial R_{C}^{2}$ | | | | | | |
| 0.26 | - | -5.9644117305 | -5.9901385315 | -6.0156285879 | -6.0408895214 | -6.0659288956 | - |
| 0.28 | - | -5.9642527298 | -5.9899791882 | -6.0154688714 | -6.0407294004 | -6.0657683380 | - |
| 0.30 | - | -5.9640937307 | -5.9898198466 | -6.0153091566 | -6.0405692810 | -6.0656077819 | - |
| 0.32 | - | -5.9639347333 | -5.9896605065 | -6.0151494433 | -6.0404091632 | -6.0654472274 | - |
| 0.34 | - | -5.9637757375 | -5.9895011681 | -6.0149897315 | -6.0402490469 | -6.0652866744 | - |
| 0.36 | - | -5.9636167434 | -5.9893418312 | -6.0148300214 | -6.0400889321 | -6.0651261228 | - |
| 0.38 | - | -5.9634577509 | -5.9891824960 | -6.0146703128 | -6.0399288189 | -6.0649655728 | - |
|  | $\partial^{2}PP(V,P_{I})/\partial R_{C}\partial R_{S}$ | | | | | | |
| 0.26→0.28 | 0.1092978607 | 0.2682985439 | 0.4276417911 | 0.5873582410 | 0.7474791914 | 0.9080367619 | - |
| 0.28→0.30 | 0.1092872765 | 0.2682863169 | 0.4276279782 | 0.5873428535 | 0.7474622521 | 0.9080183190 | - |
| 0.30→0.32 | 0.1092766922 | 0.2682741069 | 0.4276141539 | 0.5873274574 | 0.7474453156 | 0.9079998648 | - |
| 0.32→0.34 | 0.1092661080 | 0.2682618913 | 0.4276003352 | 0.5873120614 | 0.7474283734 | 0.9079814106 | - |
| 0.34→0.36 | 0.1092555152 | 0.2682496699 | 0.4275865166 | 0.5872966682 | 0.7474114284 | 0.9079629535 | - |
| 0.34→0.36 | 0.1092449281 | 0.2682374515 | 0.4275726866 | 0.5872812665 | 0.7473944862 | 0.9079444993 | - |
|  | $\partial R_{C}/\partial R_{S}$ | | | | | | |
| 0.26→0.28 | - | 0.0183250026 | 0.0447900399 | 0.0710884631 | 0.0972304226 | 0.1232258413 | - |
| 0.28→0.30 | - | 0.0183237166 | 0.0447891902 | 0.0710880544 | 0.0972304526 | 0.1232263104 | - |
| 0.30→0.32 | - | 0.0183224304 | 0.0447883432 | 0.0710876437 | 0.0972304811 | 0.1232267800 | - |
| 0.32→0.34 | - | 0.0183211442 | 0.0447874952 | 0.0710872339 | 0.0972305096 | 0.1232272486 | - |
| 0.34→0.36 | - | 0.0183198564 | 0.0447866462 | 0.0710868240 | 0.0972305386 | 0.1232277168 | - |
| 0.34→0.36 | - | 0.0183185696 | 0.0447857977 | 0.0710864123 | 0.0972305661 | 0.1232281854 | - |
| ^a^ Unless otherwise indicated, $\beta=$ 0.9, $R_{B}=$ 3.74%, $E_{G}=$ 37.49, $E_{C}=$ 169.62, $c_{G}=$ 0.016, $c_{C}=$ 0.038, $\delta=$ 0.85, $\sigma_{G}=$ 0.2404, $\sigma_{C}=$ 0.3404, $cap=$ 2.20%, and $\alpha=$ 0.181. The shaded areas illustrate the optimal values, and the comparative statics are evaluated at the optimal $R=$ 3.70%. | | | | | | | |

| **Table 8** | | | | | | | |
| --- | --- | --- | --- | --- | --- | --- | --- |
| Responsiveness of the optimal $R_{C}$ to $\alpha$.^a^ | | | | | | | |
|  | $(R_{C}(\%), L)$ | | | | | | |
| $\alpha$ | (4.80, 321) | (5.00, 320.8) | (5.20, 320.4) | (5.40, 319.8) | (5.60, 319) | (5.80, 318) | (6.00, 316.8) |
|  | $PP(V,P_{I})$ | | | | | | |
| 0.151 | 522.0004615671 | 522.6587127215 | 523.0869656460 | 523.2842230071 | 523.2494963321 | 522.9818056711 | 522.4801792534 |
| 0.161 | 526.3388524657 | 527.0007940279 | 527.4299536675 | 527.6253237682 | 527.5859057813 | 527.3107098972 | 526.7987547151 |
| 0.171 | 530.7833123598 | 531.4490226389 | 531.8790987706 | 532.0725225774 | 532.0282851536 | 531.7453865483 | 531.2228354483 |
| 0.181 | 535.3377699865 | 536.0073301896 | 536.4383330034 | 536.6297493969 | 536.5805598127 | 536.2897538620 | 535.7563300222 |
| 0.191 | 540.0063486719 | 540.6798430703 | 541.1117832160 | 541.3011289180 | 541.2468496574 | 540.9479242956 | 540.4033407878 |
| 0.201 | 544.7933784402 | 545.4708945473 | 545.9037831858 | 546.0909926829 | 546.0314812327 | 545.7242166191 | 545.1681759455 |
| 0.211 | 549.7034090386 | 550.3850378003 | 550.8188866595 | 551.0038921234 | 550.9390007576 | 550.6231689237 | 550.0553625258 |
|  | $\partial^{2}PP(V,P_{I})/\partial R_{C}^{2}$ | | | | | | |
| 0.151 | - | -5.7499557439 | -5.7748890875 | -5.7996009006 | -5.8240996523 | -5.8483939159 | - |
| 0.161 | - | -5.8195480638 | -5.8447384715 | -5.8697021908 | -5.8944474299 | -5.9189824499 | - |
| 0.171 | - | -5.8908536852 | -5.9163081221 | -5.9415307649 | -5.9665295385 | -5.9913123667 | - |
| 0.181 | - | -5.9639347333 | -5.9896605065 | -6.0151494433 | -6.0404091632 | -6.0654472274 | - |
| 0.191 | - | -6.0388563169 | -6.0648610932 | -6.0906240665 | -6.1161525280 | -6.1414536512 | - |
| 0.201 | - | -6.1156867124 | -6.1419785378 | -6.1680236812 | -6.1938290829 | -6.2194015038 | - |
| 0.211 | - | -6.1944975625 | -6.2210848819 | -6.2474207407 | -6.2735117055 | -6.2993640994 | - |
|  | $\partial^{2}PP(V,P_{I})/\partial R_{C}\partial\alpha$ | | | | | | |
| 0.151→0.161 | 1.8452039081 | 0.4533575117 | -0.9436301690 | -2.3456559726 | -3.7526115252 | -5.1643822039 | - |
| 0.161→0.171 | 1.8843584817 | 0.4582460539 | -0.9731469586 | -2.4097184401 | -3.8513606121 | -5.2979589493 | - |
| 0.171→0.181 | 1.9249620166 | 0.4633410537 | -1.0037066336 | -2.4760802015 | -3.9536726949 | -5.4363699082 | - |
| 0.181→0.191 | 1.9670976404 | 0.4686659685 | -1.0353457662 | -2.5448382308 | -4.0597055265 | -5.5798340026 | - |
| 0.191→0.201 | 2.0108543192 | 0.4742464097 | -1.0681024817 | -2.6160947763 | -4.1696258758 | -5.7285829272 | - |
| 0.201→0.211 | 2.0563273100 | 0.4801103073 | -1.1020165753 | -2.6899577654 | -4.2836102164 | -5.8828621291 | - |
|  | $\partial R_{C}/\partial\alpha$ | | | | | | |
| 0.151→0.161 | - | 0.3209074974 | 0.0785049730 | -0.1627060526 | -0.4027499721 | -0.6416482164 | - |
| 0.161→0.171 | - | 0.3237980787 | 0.0784031751 | -0.1657915388 | -0.4088115924 | -0.6506795120 | - |
| 0.171→0.181 | - | 0.3267713169 | 0.0783159099 | -0.1689306466 | -0.4149950462 | -0.6599009454 | - |
| 0.181→0.191 | - | 0.3298321877 | 0.0782458318 | -0.1721230330 | -0.4213022929 | -0.6693167666 | - |
| 0.191→0.201 | - | 0.3329859519 | 0.0781957579 | -0.1753683153 | -0.4277353719 | -0.6789314245 | - |
| 0.201→0.211 | - | 0.3362381702 | 0.0781686722 | -0.1786660740 | -0.4342964149 | -0.6887495869 | - |
| ^a^ Unless otherwise indicated, $\beta=$ 0.9, $R_{B}=$ 3.74%, $E_{G}=$ 37.49, $E_{C}=$ 169.62, $c_{G}=$ 0.016, $c_{C}=$ 0.038, $\delta=$ 0.85, $\sigma_{G}=$ 0.2404, $\sigma_{C}=$ 0.3404, $cap=$ 2.20%, and $R_{S}=$ 0.32%. The shaded areas illustrate the optimal values, and the comparative statics are evaluated at the optimal $R=$ 3.70%. | | | | | | | |

| **Table 9-1** | | | |
| --- | --- | --- | --- |
| The total effects of $cap$ on $R+R_{C}$: according to Eq. (8).^a^ | | | |
| $cap$(%) | $\partial R/\partial cap\vert_{S}$ | $\partial R_{C}/\partial cap\vert_{PP}$ | $\partial R/\partial cap$ |
| 1.90→2.00 | 0.0055426959 | 0.2505827862 | 0.2561254821 |
| 2.00→2.10 | 0.0055555804 | 0.2505578978 | 0.2561134782 |
| 2.10→2.20 | 0.0055684517 | 0.2505327167 | 0.2561011684 |
| 2.20→2.30 | 0.0055814168 | 0.2505073255 | 0.2560887423 |
| 2.30→2.40 | 0.0055942611 | 0.2504813912 | 0.2560756523 |
| 2.40→2.50 | 0.0056071991 | 0.2504554122 | 0.2560626113 |
| ^a^ The comparative statics evaluated at the optima $R=$ 3.70% and $R_{C}=$ 5.40%. | | | |

| **Table 9-2** | | | |
| --- | --- | --- | --- |
| The total effects of $R_{S}$ on $R+R_{C}$: according to Eq. (8).^a^ | | | |
| $R_{S}$(%) | $\partial R/\partial R_{S}\vert_{S}$ | $\partial R_{C}/\partial R_{S}\vert_{PP}$ | $\partial R/\partial R_{S}$ |
| 0.26→0.28 | 0.0015809059 | 0.0710884631 | 0.0726693690 |
| 0.28→0.30 | 0.0015811130 | 0.0710880544 | 0.0726691674 |
| 0.30→0.32 | 0.0015811548 | 0.0710876437 | 0.0726687985 |
| 0.32→0.34 | 0.0015816964 | 0.0710872339 | 0.0726689303 |
| 0.34→0.36 | 0.0015817359 | 0.0710868240 | 0.0726685599 |
| 0.34→0.36 | 0.0015819442 | 0.0710864123 | 0.0726683565 |
| ^a^ The comparative statics evaluated at the optima $R=$ 3.70% and $R_{C}=$ 5.40%. | | | |

| **Table 9-3** | | | |
| --- | --- | --- | --- |
| The total effects of $\alpha$ on $R+R_{C}$: according to Eq. (8).^a^ | | | |
| $\alpha$ | $\partial R/\partial\alpha\vert_{S}$ | $\partial R_{C}/\partial\alpha\vert_{PP}$ | $d(R+R_{C})/d\alpha$ |
| 0.151→0.161 | 0.0062133058 | -0.1627060526 | -0.1564927468 |
| 0.161→0.171 | 0.0063600316 | -0.1657915388 | -0.1594315072 |
| 0.171→0.181 | 0.0065053213 | -0.1689306466 | -0.1624253253 |
| 0.181→0.191 | 0.0066488546 | -0.1721230330 | -0.1654741784 |
| 0.191→0.201 | 0.0067903011 | -0.1753683153 | -0.1685780142 |
| 0.201→0.211 | 0.0069293210 | -0.1786660740 | -0.1717367530 |
| ^a^ The comparative statics evaluated at the optima $R=$ 3.70% and $R_{C}=$ 5.40%. | | | |
